# There may be many ways to be an herbivorous fish, but it usually takes a lot of guts

**DOI:** 10.1098/rsbl.2026.0467

**Published:** 2026-10-21

**Authors:** Aaron M. Davis, Delson Hays, Matthew C. Le Feuvre, Ricardo Betancur-R

**Affiliations:** ^1^ Centre for Tropical Water and Aquatic Ecosystem Research (TropWATER), James Cook University, Townsville, Queensland, Australia; ^2^ Marine Biology Research Division, Scripps Institution of Oceanography, University of California San Diego, La Jolla, CA, USA; ^3^ Jacobs Australia Pty Ltd, Melbourne, Victoria, Australia; ^4^ Department of Biology, University of Oklahoma, Norman, OK, USA

**Keywords:** adaptive radiation, ecomorphology, herbivory, integration, many-to-one, convergence

## Abstract

Classic examples of convergent trophic evolution in fishes have focused predominantly on adaptations in craniofacial morphology to piscivorous and durophagous diets. With the physiological demands of nutrient-poor, plant-based diets, adaptation to herbivory also significantly influences digestive physiology and morphology, so convergence onto similar herbivorous phenotypes in elements of postcranial morphology such as body shape and intestinal length might also be expected. Here, we integrate dietary and ecomorphological data, geometric morphometrics and phylogenetic comparative methods to investigate convergence in the body plan of Terapontoidei, an ecomorphologically diverse marine and freshwater fish radiation. We show that while diet has shaped morphology, herbivores have not converged towards distinct morphotypes, instead encompassing much of the broader body shape and size morphospace of Terapontoidei fishes. Significantly longer intestines have, however, remained a constant selective demand for adapting to plant-based diets.

## 1. Introduction

The evolution of herbivory has profoundly influenced aquatic food web function [1,2]. Despite the taxonomic frequency of herbivory among mammals (>25%) [3], its occurrence is relatively restricted (2–5% of species) in other vertebrates [1]. Contrary to the enormous diversity of fish diets, adopting plant-based diets is a rare evolutionary phenomenon, limited to a few teleost families [1,4]. One germane ecomorphological adaptation to herbivorous diets relates to intestinal length. With high energetic maintenance costs [2], the size and performance of the digestive system should be matched to food quality, with increased gut length a long-posited (but still debated) convergent character across often distantly related herbivorous fishes [4–6].

Phenotypic integration, across multiple aspects of the multivariate trait complex defining an organism's phenotype, is recognized as facilitating exploitation of previously inaccessible ecological space [7,8]. Convergent integration in fish craniofacial anatomy, body shape and size in facilitating piscivory and molluscivory has received considerable research attention [9–11]. With intestinal length a critical functional trait in processing acquired food, its evolution would similarly be predicted to covary with diet and other aspects of morphology, such as body shape. Several body-shape adaptions are recently proposed to facilitate accommodation of longer herbivore intestines, including deeper, ‘stouter’ body plans [12,13], and larger abdominal cavities [14,15] and body sizes [16,17].

The percomorph fishes of the suborder Terapontoidei (Eupercaria: Centrarchiformes) are a compelling model clade for examination of correlated intestinal length, body size/shape and dietary convergence. With eight families evolving across diverse temperate–tropical marine and freshwater ecosystems, collective lineages exhibit diverse diets, including multiple, independent evolutions of specialized herbivory [18,19]. In line with predictions of digestive theory, we test the role of herbivorous diets in shaping Terapontoidei morphology, specifically if intestinal length, body size and shape exhibit significant covariation with diet, and whether these traits converge across the multivariate adaptive landscape.

## 2. Material and methods

### (a) Phylogenomic and divergence time estimation

To reconstruct a robust phylogenetic framework for Terapontoidei, we assembled 34 genomic datasets using *MEGAHIT* v1.2.9 [20] from new genomic, transcriptomic and exon-capture data, combined with five published terapontoid and three centrarchoid outgroup genomes (supplementary material, table S1). A total of 1133 exonic markers were extracted following Hughes *et al.* [21], aligned with *MACSE* v2.08 [22], and concatenated with *AMAS* [23]. Taxon sampling was expanded to 109 species using published data for 12 anchor mitochondrial genes and the nuclear genes RAG1-2 (supplementary material, table S1). For the full alignment and three balanced 385-gene subsets (supplementary material, table S2), we inferred concatenation-based maximum-likelihood trees in RAxML-NG [24] under GTR+G with codon partitioning, and multispecies coalescent trees in *ASTRAL* v5.7.1 [25] from gene trees estimated in *IQ-TREE* v2.2.2.6 [26]. Two COI-only taxa (*Parascorpis typus*, *Neoscorpis lithophilus*) showed unstable placement but were retained, as their exclusion did not alter downstream inferences. Divergence times were estimated by ‘congruifying’ each of the eight focal trees (four ML, four ASTRAL) to a time-calibrated Eupercarian reference backbone [27], with node calibrations generated in *congruify.phylo* [28] and applied in *treePL* [29]. Unless otherwise noted, presented analyses used the all-gene concatenation ML tree, but were repeated across the eight-tree set to account for phylogenetic uncertainty.

### (b) Anatomical preparation and trait quantification

Lateral body shape of Terapontoidei specimens (sourced from survey, museum and personal collections) was quantified via digitization of the Cartesian coordinates of 17 landmarks placed on specimen photographs (supplementary material, figure S1; 1–4 specimens per species, mean = 2.1) using *TpsDig* 2.17 [30]. A generalized least squares Procrustes superimposition of landmark data (GPA) was conducted in *geomorph* [31], with species’ mean shapes calculated before subsequent analyses, and subjected to principal component analysis (PCA).

Digestive tracts from some specimens were dissected out and intestinal length measured as the distance (in mm) from the pyloric outlet of the stomach to the rectum (1–36 specimens, mean = 6.5; supplementary material, table S3). Often, the same specimen was used for external and internal morphometrics, but this was not always possible (e.g. specimen warping, poor preservation). Because of limitations on specimen availability for destructive dissection, intestinal lengths from two *Girella* species were derived from literature values from similarly sized specimens [32]. Species' maximum standard length (mm) was also collated from literature datasets. To minimize allometric biases in intestinal length, we performed phylogenetic size correction (lambda (*λ*) optimization [33]) by regressing each species' averaged intestinal length against averaged standard length (both log_10_-transformed) of the processed specimens from which we directly measured intestinal length. All downstream analyses on species' intestinal length used resultant residual values.

### (c) Dietary classification and quantification

For analyses requiring categorical classification of diet species' proportionate dietary data (derived from personal and literature-sourced volumetric stomach content analyses; supplementary material, table S3) was coded according to each species' degree of herbivory: ‘carnivore’ (<20% plant-autotrophic diet), ‘omnivore’ (plant-autotrophic diet >20% and <80%) and ‘herbivore’ (>80% plant-autotrophic diet [3]). Although no species are classified as detritivores, we follow previous examples [15] in combining plant-detrital material as herbivorous–detritivorous diets probably face similar selective pressures to digest recalcitrant diets. Downstream analyses requiring quantitative dietary traits, rather than discrete categorizations, used logit-transformed proportion [34] of plant material as input. Although we acknowledge that imposing categorical dietary thresholds and trophic regimes (required for some analyses) can separate species with very similar diets, we also use a subsequent suite of quantitative and purely data-driven analyses (with no *a priori* dietary classifications) to develop an integrative view of Terapontoidei ecomorphological evolution.

### (d) Testing for ecomorphological correlations

Differences in intestinal length between dietary categories (carnivore, omnivore and herbivore) were conducted with univariate phylogenetic ANOVAs using species' intestinal length residuals and a Brownian motion (BM) phylogenetic covariance matrix (10 000 iterations) in the R package *RRPP* [35]. Differences in overall multivariate body shape (GPA landmarks) of trophic guilds were conducted via multivariate ANOVA and subsequent *post hoc* pairwise tests. This analysis compares overall body-shape variation but does not identify which portions of the body are the most variable between groups. To potentially identify those traits, we performed additional phylogenetic ANOVAs and *post hoc* pairwise tests on a subset of cranial landmarks (nos. 1–4 and 15–17), trunk landmarks (nos. 5, 11–14), and caudal peduncle landmarks (nos. 6–10), respectively (see supplementary material, figure S1).

We then used partial least squares (PLS) correlation analysis [31] followed by *compare.pls* [36] to quantify and compare the level of integration between Terapontoidei morphology and diet. The PLS analytical suite accommodates both complex, multi-dimensional traits (e.g. shape) and univariate data (body and intestinal length) and is robust to differing sample and variable sizes. Specific traits compared in PLS analyses were: intestinal length (residuals) versus diet (logit-transformed proportion of plant material); total body shape (all Procrustes-aligned species landmarks) versus diet; total body shape versus intestinal length; species maximum standard length (log-transformed) versus diet; species maximum standard length versus intestinal length; log-transformed species’ centroid sizes (square root of the sum of squared distances of all digitized specimen landmarks from their centroid) versus diet; and centroid size versus intestinal length. Owing to the high number of resultant univariate and pairwise tests, a post-analysis Benjamini–Hochberg false discovery rate *p*-value adjustment was used.

### (e) Evolutionary modelling

We also performed evolutionary model comparisons to examine the role of diet in shaping intestinal length, body shape (PC axes 1–4, selected according to broken-stick criterion; supplementary material, table S4) and maximum body length in *mvMORPH* [37] using SIMMAP ancestral character state reconstruction based on an ER (equal rates) model of trait change selected from earlier model testing of the best fit of discrete ‘herbivore’ or ‘non-herbivore’ tip states (supplementary material, table S5). We fitted two single-regime and two multi-regime models of trait evolution: (i) a BM diet-independent model (BMDI), (ii) an Ornstein–Uhlenbeck (OU) diet-independent model (OUDI), (iii) a BM diet-dependent model (BMDD), (iv) an OU diet-dependent model (OUDD), (v) and an additional early-burst ACDC (accelerating–decelerating) model to capture any early phenotypic diversification expected under adaptive radiation. Model fit was run over 100 SIMMAP stochastic character maps simulated on each tree in our eight-tree set, and compared using Akaike information criterion weights (AIC_w_) averaged across the eight trees. Because PCA-based model fitting may bias support towards more complex evolutionary models [38], we appraised whether our analytical pipeline could correctly recover a simpler generating process by simulating a trait (constrained to our PC 1–4 data range) evolving under strict BM and replicated model analysis on our consensus tree. To appraise whether our PCA treatment of body shape (required for modelling and convergence analyses) was relevant to earlier use of raw GPA landmarks in ecomorphological correlations, we also compared PLS results based on PCA versus raw GPA landmarks (supplementary material, table S6).

### (f) Convergence analyses

Given various phylogenetic convergence analyses often have differing computational demands and assumptions (particularly around *a priori* selective regime definition) [39], we used several analyses in concert to assess the magnitude of convergence. We first used the recently developed *CalconvCt* function from the *convevol* package v2.0.0 [40,41], as well as the *search.conv* function implemented in the *RRPhylo* package [42]. Using the *convevol C*_t_ distance-based metrics, positive *C*_t_ values indicate different degrees of convergence up to 1 (complete convergence), whereas 0 and negative values indicate a lack of convergence [41]. We tested six *a priori* ‘herbivore’ groupings (based on our earlier species dietary categorical classification and combination of some ‘sister’ herbivore taxa into single groups: supplementary material, figure S2) to avoid uninformative comparisons (i.e. ‘herbivores’ across (i) *Kyphosus–Neoscorpis*, (ii) *Girella*, (iii) *Syncomistes–Scortum*, (iv) *Pingalla–Bidyanus*, (v) *Medialuna*, and (vi) *Helotes*). Statistical significance was tested by comparing the observed *C*_t_ measures of the first four PC axes of body shape evolution with a null distribution of simulated BM evolution (*n* = 100).

The computationally efficient *search.conv* [42] function tests morphological convergence by assessing the angle (*θ*_real_) between phenotypic vectors (vectors of PC scores) between species and comparing this angle with a random BM expectation. Possible angle values range from 0° (similar phenotypes) to 180° (contrasting phenotypes) [43]. We applied *search.conv* to test for morphological convergence in ‘auto’ mode (no pre-defined converging herbivorous clades), and then also for species evolving *a priori* defined herbivorous dietary habits (as per *convevol* analyses). Both these analyses can accommodate complex, multi-dimensional data, but not univariate traits (i.e. intestinal length).

To identify potential convergence without *a priori* designation of trophic regimes, we also used the *ℓ1ou* package [44] to assess the first four PC axes resulting from previous body shape PCA analyses, and univariate intestinal length residual data and maximum species standard length. Convergent optima were identified using AIC_C_, but also the phylogenetically informed Bayesian information criterion (pBIC), a more conservative parameter option that guards against false positives while retaining reasonable precision [44]. To determine whether scale of identified convergence could have occurred by chance under a non-convergent process [45], results were compared with null distributions generated by simulating two simpler evolutionary models 99 times: a constant-rate BM model and a single-regime OU process. All phylogenetic analyses were performed using R version 2.15 [46], with data manipulations conducted using functions from *phytools* [47].

## 3. Results

### (a) Phylogenetic analyses

Genome-scale phylogenies recovered a mixture of stable and uncertain relationships within Terapontoidei. Three genera (*Atypichthys*, *Neoscorpis* and *Parascorpis*) showed unstable placement, resolving alternatively as deeply nested or early-branching lineages. By contrast, the sister relationship between Kuhliidae and Terapontidae was recovered in all trees [19,48], and most trees (5/8) supported a broader clade excluding a scorpidid-girellid clade. Families were otherwise largely monophyletic. Divergence-time estimates were broadly similar to Davis & Betancur [19], although some key nodes differed modestly (e.g. crown Terapontidae approx. 37–38 Ma versus approx. 33 Ma), probably reflecting the denser calibration framework of previous Eupercarian backbones [27] used here.

### (b) Morphometric analyses

Intestinal length (corrected for body size) varied considerably across the Terapontoidei. Relative intestinal length (RIL: intestinal length/standard length; a commonly used ecomorphological metric), for example, ranged from ≤1.0 in several genera (*Graus*, *Kuhlia*, *Terapon*, *Pelates* and *Pelsartia*; supplementary material, figure S2 and table S3), up to ≥4–5 (*Girella*, *Kyphosus*, *Scortum* and *Syncomistes*), aligning closely with earlier studies across specific clades [49,50]. Aligning with digestive theory predictions, carnivores generally had the shortest intestines, omnivores intermediate and herbivores the longest intestinal lengths (figure 1b). *RRPP* ANOVA revealed significant differences in intestinal length between these broad trophic designations (*F* = 35.88, *R*^2^ = 0.44, effect size (*Z*) = 6.1, *p* < 0.001). Subsequent *post hoc* pairwise tests showed significant differences between intestinal lengths of herbivores and all other trophic designations, but not between carnivores and omnivores (carnivore:herbivore, *Z* = 4.10, *p* < 0.001; carnivore:omnivore, *Z* = 1.41, *p* = 0.07; omnivore:herbivore, *Z* = 3.89, *p* < 0.001).

**Figure 1.**
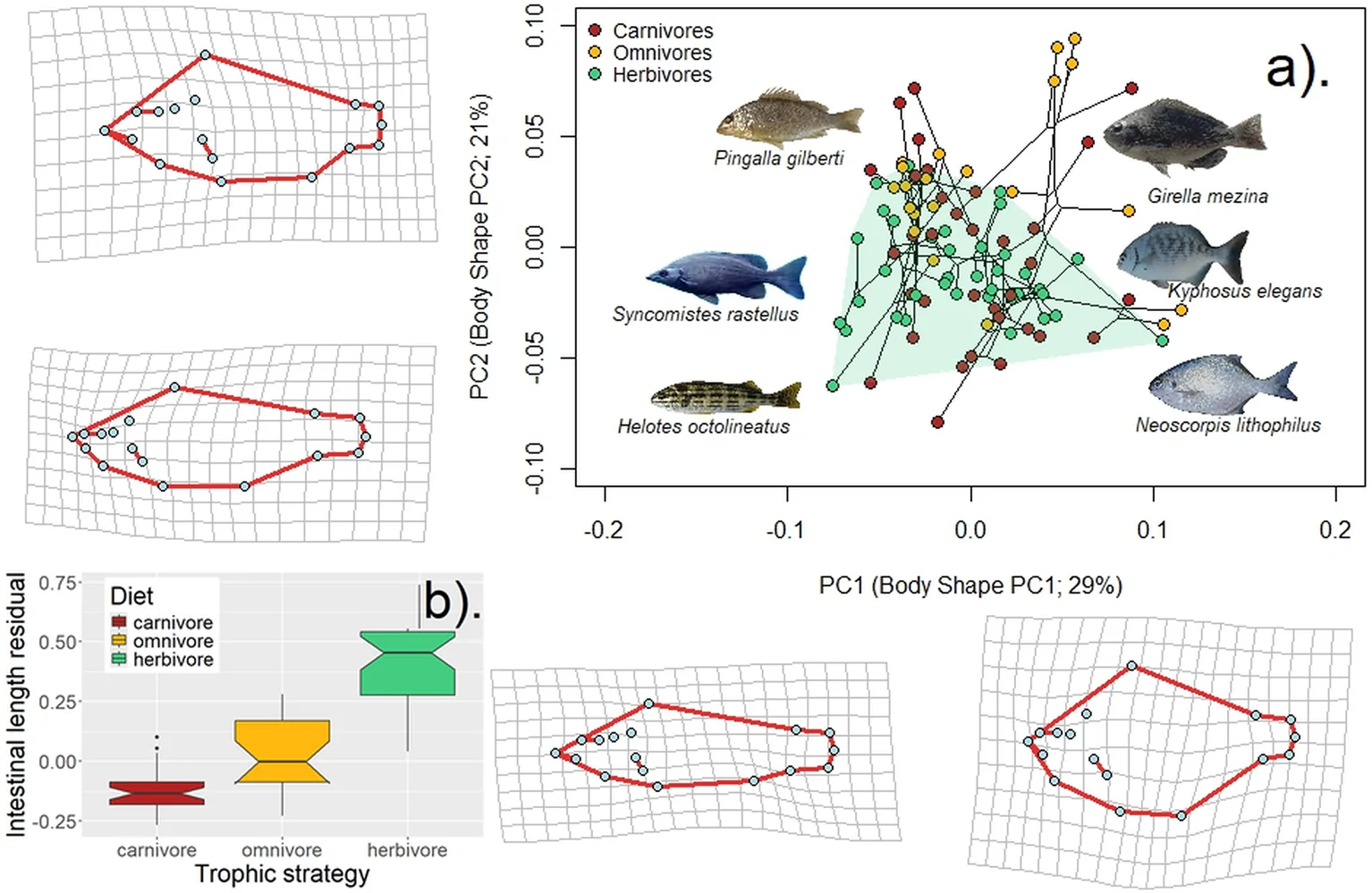
(a) Projection of the Terapontoidei phylogenetic tree into the lateral view PC morphospace (PC1, PC2). Thin-plate spline deformation grids represent body-shape extremes along first and second PC axes (relative to mean specimen shape). Species are colour-coded according to trophic categories (supplementary material, table S3). Polygon indicates herbivorous morphospace. Images of several specialized herbivorous species occurring near PC extremes are identified. (b) Boxplots depicting species phylogenetically and size-corrected intestinal length variation colour-coded according to trophic categories.

The Terapontoidei also display considerable morphological diversity in body shape, with PCA identifying four significant major axes explaining most of the variation (cumulative variation 73%; figure 1a; supplementary material, table S4). These axes primarily are related to: body depth (PC1; compressiform/ovate versus elongate/fusiform body plans; 29% variation), shifts towards shorter caudal peduncles, and larger cranial/anterior bodies (PC2; 21%), variation in eye size and maxilla lengths (PC3; 15%), and posterior versus anterior dorsal and anal fin insertion (PC4; 8%). There was, however, considerable overlap across trophic categories across the main axes of phylomorphospace.

### (c) Ecomorphological correlations

*RRPP* tests of difference in average body shape across different trophic categories revealed no significant differences across carnivorous, omnivorous and herbivorous dietary strategy classifications (*F* = 1.68, *Z* = 1.40; *p* = 0.08). Nor were there any significant differences between head shape (*F* = 1.37, *Z* = 0.86; *p* = 0.19), trunk shape (*F* = 1.37, *Z* = 0.85; *p* = 0.18) or caudal shape (*F* = 0.71, *Z* = −0.15; *p* = 0.55) according to trophic category (all pairwise tests presented in supplementary material, table S7).

PLS analyses (supplementary material, table S8 and figure S3) revealed significant correlation (i.e. significant *r*_PLS_ scores) between both body shape, intestinal length and logit-transformed proportion of plant material in diet. Body size (as either species' maximum standard length or analysed specimen centroid size) had no significant correlation with either plant material in diet or intestinal length. Pairwise comparisons of standardized effect sizes across all traits (z-scores), however, revealed that while dietary habits were most strongly correlated with intestinal length, they did not show significantly higher levels of covariation with diet than body shape on the consensus ML tree (*p* = 0.17).

Of the separate body shape module PC axes (cranial, trunk and caudal peduncle shape), cranial shape was the only body shape module subdivision that returned a significant level of integration with diet (supplementary material, figure S4). Pairwise comparison of standardized effect sizes across intestinal length and all shape modules (*z*-scores) revealed no significant differences between intestinal length and the head shape module and relationship to diet. All PLS analyses (gross morphology and body shape modules) were broadly consistent when conducted across the eight-tree set (supplementary material, tables S9 and S10).

### (d) Evolutionary modelling

Evolutionary model comparisons of the potential role of diet in shaping morphology identified overwhelming support for an OUDD model for both intestinal length and overall body shape (AIC_W_, approx. 1.0; supplementary material, table S11). Evolutionary modelling of maximum body length revealed similar support for both an OUDI (AIC_W_ 0.53) and an OUDD model (AIC_W_ 0.47), with minimal support for alternatives. Our simulated BM dataset suggested that our analytical pipeline had the capacity to correctly identify simpler generating evolutionary models over more complex alternatives (supplementary material, table S12).

### (e) *Convevol* analysis

*C*_t_ analyses of multivariate body shape (supplementary material, table S13) identified non-significant values across *C*_t_-1 to *C*_t_-3 metrics (*C*_t_-1 = −0.056, *p* = 0.25; *C*_t_-2 = −0.002, *p* = 0.2; *C*_t_-3 = −0.015, *p* = 0.14), indicating that herbivorous lineages have not converged phenotypically and may instead be slightly more divergent than expected under BM. Although *C*_t_-4 (overall weighted) was statistically significant (*C*_t_-4 = 0.001, *p* = 0.01), its effect size was effectively zero, providing no evidence of biologically meaningful convergence in overall body shape among herbivores.

### (f) *RRphylo* analyses

The analyses of *search.conv* failed to detect morphological convergence in collective body shape PCs under the default ‘auto-clade’ mode. Assessing convergence using the pre-defined herbivorous trophic categories identified a mean angle between phenotypic vectors of all herbivores of 82.55° (*p* = 0.008), but with a time–distance‐standardized *θ*_real_ of 1.52, not reaching statistical significance (*p* = 1.00). Although herbivorous species are more similar in multivariate body shape than expected under a BM model, the relatively large mean angle (approx. 82°) indicates modest phenotypic similarity, and herbivores overlap extensively with non-herbivores in morphospace (figure 2). Results were highly consistent across the eight-tree set (supplementary material, table S14).

**Figure 2.**
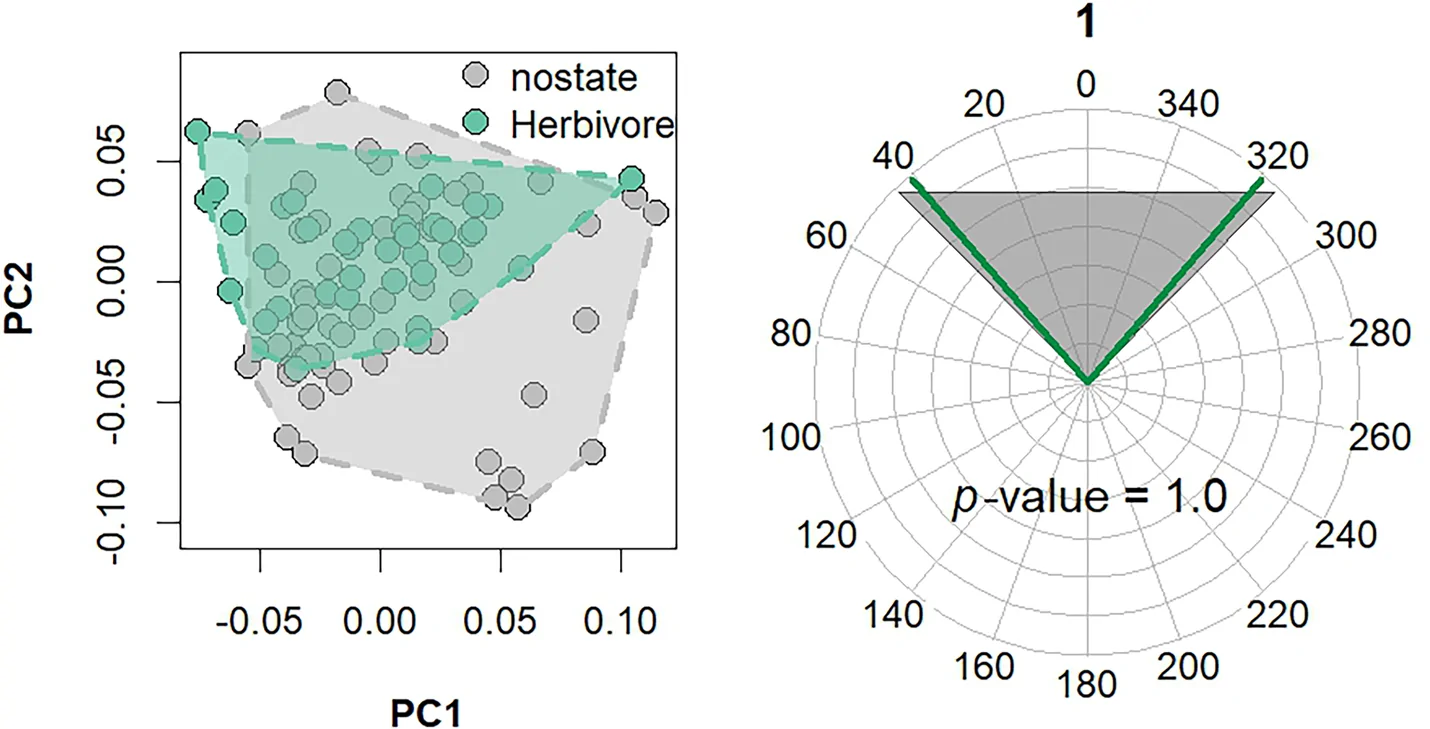
*RRphylo* body-shape convergence across Terapontoidei clades according to herbivory versus non-herbivory (species belonging to different states indicated by coloured convex hulls). Circular plot represents mean *θ*_real_ angle between herbivorous species (green lines) compared with the random distribution of angles obtained by shuffling states across species in the tree (*θ*_random_; grey-shaded area).

### (g) *ℓ1ou* analyses

For body shape PCs 1–4, *ℓ1ou* analyses (using AIC_C_) identified 12 total shifts in trait optima (AIC_C_ = −1753.8), with 6 collapses onto 3 convergent optima (AIC_C_ = −1761.6) and 6 non-convergent optima. Convergent clade examples included single species within the freshwater *Scortum* and *Syncomistes*, marine *Kyphosus* and *Girella*, and *Scortum* and *Kuhlia* evolving similar body shapes (supplementary material, figure S5). The *ℓ1ou* model using pBIC for shift detection on body shape PCs identified six unique body-shape optima (AIC_C_ = −1677.1), mainly comprised the Kyphosidae and a unique optimum, including the majority of Kuhliidae and Terapontidae, none of which collapsed to convergent regimes. Convergence in maximum body size was similarly limited, with AIC_C_ analyses identifying nine shifts (AIC_C_ = −95.86), collapsing onto six regimes (AIC_C_ = −103.15), with collapses onto different convergent smaller body size optima for the Microcanthidae and marine Terapontidae, some smaller *Kuhlia* and *Pingalla*, and *Kuhlia* and *Labracoglossa* (supplementary material, figure S6). pBIC identified two shifts (pBIC = −51.5) that converged (pBIC = −53.3), involving all Kuhliidae and Terapontidae, regardless of diet. Thus, convergence in body shape and size was limited, sensitive to model stringency, and unrelated to diet.

For intestinal length, the *ℓ1ou* model using AIC_C_ for shift detection identified 14 distinct adaptive shifts from mean trait values (AIC_C_ = −147.52), which collapsed to 6 distinct regimes, composed of 13 shifts to convergent peaks and 1 unique non-convergent peak shift (AIC_C_ = −178.52). Several independent marine herbivore (*Medialuna*, several *Kyphosus*–*Neoscorpis, Girella*) and freshwater (*Leiopotherapon aheneus*) omnivore lineages converged onto a similar phenotypic optimum (figure 3a). Some independent herbivorous marine *Girella* and freshwater (*Syncomistes* and *Scortum*) genera with long intestines converged, as did several of the freshwater herbivores–omnivores (*Pingalla*, *Bidyanus* and *Hephaestus*), and the marine herbivore *Helotes*. Most of the longer intestinal length *Kyphosus* species evolved to a unique phenotypic optimum. Under the more conservative pBIC, five distinct adaptive shifts were identified (pBIC = −68.3), collapsing onto a single convergent regime and four unique phenotypic optima (pBIC = −69.54). Here, several highly herbivorous freshwater (*Scortum*–*Syncomistes*) and marine taxa (*Girella melanichthys*) converged onto similar long intestinal length optima (figure 3b).

**Figure 3.**
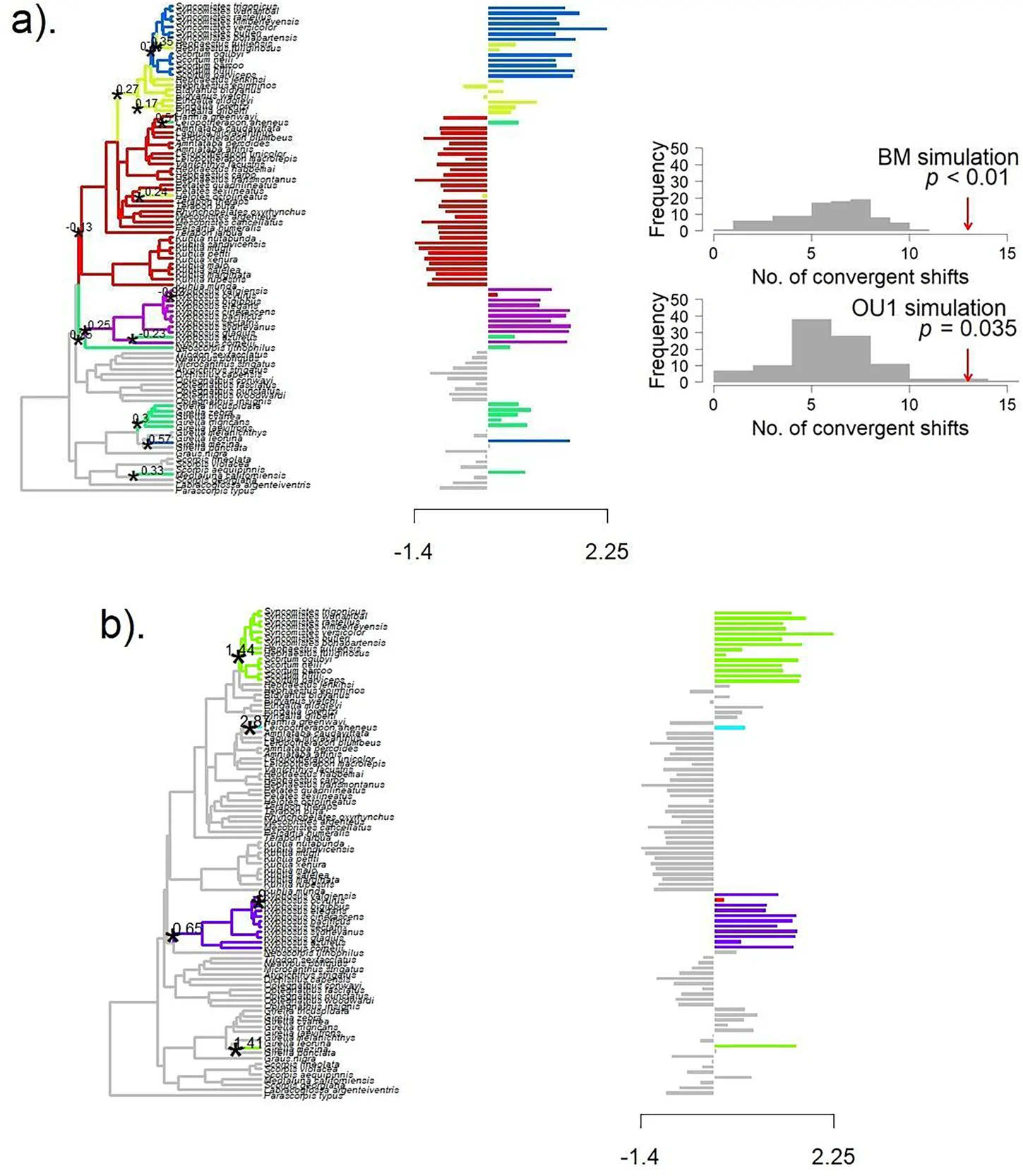
Convergent shifts in Terapontoidei morphology (intestinal length) using *ℓ1ou* under a multi-peak OU process, with regimes selected using AIC_C_ (a) and pBIC (b). Phylogenetic tree, with phenotypic peak shifts from mean trait values indicated by coloured branches, and non-convergent (grey) regimes painted onto branches (edges of the same colour are inferred to have converged). Bar graphs show the range of trait phenotypic values used in analysis. Inset in (a) shows observed extent of convergent regimes compared with the null expectation under simulated non-convergent BM and OU1 models.

Across eight alternative trees, AIC_C_-based analyses produced very similar results in terms of number of shifts, shifts collapsing to convergent regimes, the specific converging clades and total number of converging clades (supplementary material, table S15), with convergence under pBIC more limited, particularly on the Astral tree set. Comparisons of intestinal length evolution simulated 99 times under OU1 and BM null distribution models revealed that intestinal length had significantly greater numbers of convergent shifts than expected by chance (with no convergence identified for simulations using pBIC; figure 3a). Overall *ℓ1ou* results suggest that convergence onto similar adaptive phenotypic peaks in body shape and size is extremely rare for herbivores (or any trophic strategy) but does repeatedly and consistently occur for intestinal length.

## 4. Discussion

The evolutionary history of the Terapontoidei is characterized by repeated evolution of herbivory across marine and freshwater clades, paralleling the ‘iterative’ or ‘reticulate’ adaptive radiations described in other fish groups [51–53]. These repeated shifts towards herbivory have not produced strong convergence in overall body shape or size, and consistent with recent studies [13,54], our results provide limited evidence for a single body-shape optimum associated with broad trophic strategies. Herbivorous taxa range from fusiform–sagittiform morphotypes (‘sharp-nosed’ *Syncomistes* grunters) to deep-bodied, ‘short-headed’ forms (*Kyphosus* and *Neoscorpis*), with significant body-shape convergence being rare. This ‘many-to-one’ mapping of form to function [55] suggests multiple morphological solutions to similar adaptive demands, weakening covariance among traits and potentially limiting convergence in body form [56].

Paralleling other hyper-diverse radiations [57], repeated evolution of lengthened intestines represents a functional innovation evolving independently over tens of millions of years across marine and freshwater Terapontoidei. Although herbivorous and omnivorous clades display broad body-shape diversity, selection for longer intestines to increase transit time of recalcitrant plant material through the alimentary canal [3] appears a dominant constraint during trophic evolution. Contrary to recent predictions [16,17], larger body size was a poor predictor of herbivory and intestinal length within the Terapontoidei. Although specialized herbivorous genera (*Kyphosus* and *Girella*) are among the largest taxa (>600 mm SL) and possess exceptionally long intestines, other highly herbivorous genera (*Pingalla* and *Syncomistes*) rank among the smallest species in the clade (<130 mm SL) while still possessing some of the longest intestinal lengths.

Nutritional targets among nominally herbivorous fishes are difficult to define [58], and associated morphological optima for biofilm scraping, filamentous algal feeding or macrophyte consumption may differ substantially [32,58]. Several mechanisms (untested here) may also obscure evolution of distinct external ‘herbivore morphotypes’ accommodating elongated intestines, including enlargement of abdominal cavities, increased body width, increased intestinal coiling complexity, modified digesta passage rates, gastrointestinal fermentation, digestive enzyme profiles, intestinal surface area and cellular-level diversification of the intestinal epithelium accompanying herbivory [6,58–60]. Examples also exist in Terapontoidei herbivores like *Helotes* [61], exhibiting longer intestines (RIL ≥ 1.5) than closely related carnivores (*Pelates*, *Terapon*; RIL ≤ 1.0), but substantially shorter than in other highly herbivorous genera (*Kyphosus*, *Scortum* and *Syncomistes*; RIL ≥ 5.0). This demonstrates that specialized herbivory can evolve from carnivorous ancestors without extreme intestinal elongation, a pattern also observed in other fish clades [6,62].

Overall, results suggest that the physiological demands of herbivory impose recurring constraints on digestive anatomy, whereas external body morphology remains comparatively flexible. Although overall body plan probably reflects multiple selective pressures, including habitat and locomotion [13], repeated convergence in intestinal length highlights the importance of internal soft anatomy in meeting the challenges of herbivory.

## Supplementary Material



## Data Availability

All data supporting our paper (phylogenetic trees, body shape files, dietary data, genomic information and repositories) are provided in the supplementary material. Supplementary material is available online [63].
