# Supplementary material for: There may be many ways to be an herbivorous fish, but it usually takes a lot of guts
Source: Biol Lett. 2026 Oct 21;22(10):20260467. doi: 10.1098/rsbl.2026.0467 (PMC13682930; doi:10.1098/rsbl.2026.0467)
Supplement: Supplementary file 1 [file rsbl.2026.0467.zip › rsbl.2026.0467/rsbl20260467_si_001.docx]

**ELECTRONIC SUPPLEMENTARY INFORMATION**

**Figure S1** Body shape landmarks used to quantify lateral body shape in the Terapontoidei. Subdivisions of cranial module comprise 7 landmarks (red landmarks); trunk module comprising 5 landmarks (yellow); and caudal module comprising 5 landmarks (green). Landmarks specifically comprise: (1) anterior tip of snout; (2) posterior-most end of lips; (3) anterior-most and (4) posterior-most ends of orbit; (5) anterior and (6) posterior insertions of the dorsal fin; (7) upper insertion of caudal fin; (8) midpoint of the origin of caudal fin; (9) lower insertion of caudal fin; (10) posterior and anterior (11) insertions of anal fin; (12) insertion of pelvic fin; (13) lower and (14) upper insertions of pectoral fin; (15) dorsal-most end of operculum; (16) ventral tip of cleithrum; and (17) dorsal end of pre-opercular groove.

**Figure S2** Relative intestinal length (RIL) variation (intestinal length (mm)/standard length (mm) across the Terapontoidei phylogeny (ML consensus tree). Diet and RIL variation color-coded according to broad trophic categories outlined in Table S3.

**Table S3** Literature data sources and specimen numbers used in this study to describe morphology and trophic habits in fishes of the Terapontoidei (references are detailed below). * indicates literature derived values.

| Species | Proportion plant in diet | Standard length (mm) | Intestinal length (mm) (specimen number) | Relative Intestinal Length (RIL) | Maximum length (mm) | Specimen numbers (body shape) | Trophic category | Dietary References |
| --- | --- | --- | --- | --- | --- | --- | --- | --- |
| *Amniataba affinis* | 0.43 | 31.4 | 25.4 (1) | 0.81 | 185 | 1 | omnivore | Davis *et al*. (2016). |
| *Amniataba caudavittata* | 0.08 | 129 | 120 (7) | 0.93 | 246 | 2 | carnivore | Davis *et al*. (2016). |
| *Amniataba percoides* | 0.56 | 113.52 | 115.61(15) | 1.02 | 200 | 3 | omnivore | Davis *et al*. (2016). |
| *Atypichthys strigatus* | 0.104 | 133 | 145 (2) | 1.09 | 202.25 | 3 | carnivore | Glasby & Kingsford (1994). A. M. Davis, unpublished data. |
| *Bidyanus bidyanus* | 0.9 | 180 | 380 (2) | 2.11 | 493 | 3 | herbivore | Davis *et al*. (2016). |
| *Bidyanus welchi* | 0.27 | 231 | 389.05 (7) | 1.68 | 369 | 2 | omnivore | Davis *et al.* (2016). |
| *Dichistius capensis* | 0.21 | 304.5 | 282.5 (4) | 0.93 | 647.2 | 2 | omnivore | Bennett & Griffiths (1986). A. M. Davis, unpublished data. |
| *Girella cyanea* | 0.915 | 205 | 522 (4) | 2.55 | 614.84 | 2 | herbivore | Clements & Choat (1997); Clements & Zemke‐White (2008); Lewis (2012) |
| *Girella laevifrons* | 0.801 | 167 | 462 (1) | 2.77 | 309.847 | 1 | herbivore | Fuentes and Cancino (1990), A. M. Davis, unpublished data. |
| *Girella leonina* | 0.993 | 250 | 380 (1) | 1.52 | 372.14 | 1 | herbivore | Kanda & Yamaoka (1995). |
| *Girella melanichthys* | 0.99 | 278 | 500 (1) | 1.80 | 404.5 | 1 | herbivore | Kanda & Yamaoka (1995). |
| *Girella mezina* | 0.95 | 296 | 1480 * | 5.00 | 364.05 | 1 | herbivore | Kanda & Yamaoka (1995). |
| *Girella nigricans* | 0.87 | 150 | 303 (4) | 2.02 | 533.94 | 3 | herbivore | Barry & Ehret (1993); Behrens MD, Lafferty KD (2012). A. M. Davis, unpublished data. |
| *Girella punctata* | 0.95 | 270 | 513 * | 1.90 | 404.5 | 2 | herbivore | Saburomaru & Tsukahara (1984). |
| *Girella tricuspidata* | 0.959 | 249 | 655 (4) | 2.63 | 574.39 | 3 | herbivore | Russell (1983); Anderson (1986). A. M. Davis, unpublished data. |
| *Girella zebra* | 0.98 | 220 | 660 (3) | 3.00 | 436.86 | 3 | herbivore | Shepherd & Edgar (2013). A. M. Davis, unpublished data. |
| *Graus nigra* | 0.085 | 204 | 216 (2) | 1.06 | 522.614 | 2 | carnivore | Aldana *et al.* (2002); Boyle & Horn (2006); Fuentes (1982). A. M. Davis, unpublished data. |
| *Hannia greenwayi* | 0.24 | 81.2 | 74 (8) | 0.91 | 140 | 2 | omnivore | Davis *et al*. (2016). |
| *Helotes octolineatus* | 0.97 | 156.68 | 250.2 (22) | 1.60 | 229 | 2 | herbivore | Davis *et al*. (2016). |
| *Hephaestus carbo* | 0.01 | 138.07 | 135.21 (24) | 0.98 | 280 | 4 | carnivore | Davis *et al*. (2016). |
| *Hephaestus epirrhinos* | 0.2 | 226.67 | 302.69 (3) | 1.34 | 420 | 2 | carnivore | Davis *et al*. (2016). |
| *Hephaestus fuliginosus* | 0.71 | 288.8 | 620.9 (22) | 2.15 | 450 | 4 | omnivore | Davis *et al*. (2016). |
| *Hephaestus habbemai* | 0.07 | 100 | 102 (1) | 1.02 | 161.8 | 2 | carnivore | Davis *et al*. (2016). |
| *Hephaestus jenkinsi* | 0.57 | 198.92 | 423.64 (15) | 2.13 | 421 | 4 | omnivore | Davis *et al*. (2016). |
| *Hephaestus transmontanus* | 0.02 | 79.99 | 51.64 (20) | 0.65 | 127 | 2 | carnivore | Davis *et al*. (2016). |
| *Hephaestus tulliensis* | 0.75 | 160.25 | 384.59 (15) | 2.40 | 220 | 3 | omnivore | Davis *et al*. (2016). |
| *Kuhlia malo* | 0.001 | 161 | 136 (1) | 0.84 | 157 | 1 | carnivore | A. M. Davis, unpublished data. |
| *Kuhlia marginata* | 0.001 | 137 | 122 (2) | 0.89 | 180 | 3 | carnivore | Hyslop (1999); Resh *et al.* (1999), A. M. Davis, unpublished data. |
| *Kuhlia mugil* | 0.08 | 132 | 96 (5) | 0.73 | 404.5 | 3 | carnivore | Maciolek (1981); Nanake *et al*. 2011; A. M. Davis, unpublished data. |
| *Kuhlia munda* | 0.001 | 186 | 156 (1) | 0.84 | 135 | 1 | carnivore | A. M. Davis, unpublished data. |
| *Kuhlia nutabunda* | 0.001 | 95 | 85 (1) | 0.89 | 242 | 1 | carnivore | A. M. Davis, unpublished data. |
| *Kuhlia petiti* | 0.1 | 85 | 65 (1) | 0.76 | 201 | 3 | carnivore | A. M. Davis, unpublished data. |
| *Kuhlia rupestris* | 0.1 | 216 | 196 (12) | 0.91 | 364.05 | 3 | carnivore | Pusey *et al*. (2004). |
| *Kuhlia salelea* | 0.001 | 114 | 89 (1) | 0.78 | 114 | 1 | carnivore | A. M. Davis, unpublished data. |
| *Kuhlia sandvicensis* | 0.01 | 116 | 79 (2) | 0.68 | 202.25 | 2 | carnivore | Tester &Trefz (1954). |
| *Kuhlia xenura* | 0.001 | 38 | 25 (1) | 0.66 | 220 | 1 | carnivore | Tester & Takata (1953); A. M. Davis, unpublished data. |
| *Kyphosus azureus* | 0.987 | 186 | 450 (1) | 2.42 | 364.05 | 2 | herbivore | Barry & Ehret (1993); Sturm & Horn (1998). |
| *Kyphosus bigibbus* | 0.998 | 165 | 535 (1) | 3.24 | 606.75 | 2 | herbivore | Clements & Choat (1997); Clements & Zemke‐White (2008). |
| *Kyphosus cinerascens* | 0.999 | 250 | 1221 (3) | 4.88 | 530 | 3 | herbivore | Choat *et a*l. (2002). Choat et al. (2004); A. M. Davis, unpublished data. |
| *Kyphosus cornelii* | 0.997 | 238 | 1112 (4) | 4.67 | 566.3 | 3 | herbivore | Rimmer (1986). A. M. Davis, unpublished data. |
| *Kyphosus elegans* | 0.999 | 260 | 888 (1) | 3.42 | 530 | 1 | herbivore | Cruz-Escalona *et al*. (2009.) |
| *Kyphosus gladius* | 0.999 | 250 | 1202 (1) | 4.81 | 520 | 1 | herbivore | A. M. Davis, unpublished data. |
| *Kyphosus ocyurus* | 0.6 | 239 | 485 (1) | 2.03 | 566.3 | 1 | omnivore | Knudsen (2013); A. M. Davis, unpublished data. |
| *Kyphosus pacificus* | 0.999 | 218 | 995 (1) | 4.56 | 614.84 | 1 | herbivore | Disalvo et al., 2007 |
| *Kyphosus sectatrix* | 0.999 | 236 | 910 (1) | 3.86 | 760 | 2 | herbivore | Randall (1967); Kiparissis *et al.* (2012), |
| *Kyphosus sydneyanus* | 0.999 | 280 | 1400 (4) | 5.00 | 647.2 | 2 | herbivore | Clements &Choat (1997); Clements & Zemke‐White (2008); Shepherd & Edgar (2013); Russell (1983). |
| *Kyphosus vaigiensis* | 0.999 | 298 | 1198 (1) | 4.02 | 900 | 2 | herbivore | Clements & Choat (1997); Choat et al. (2004); Clements & Zemke‐White (2008). |
| *Labracoglossa argentiventris* | 0.001 | 161 | 180 (1) | 1.12 | 161.8 | 2 | carnivore | Masuda &Allen (1993). |
| *Lagusia micracanthus* | 0.02 | 55 | 46 (1) | 0.84 | 128 | 2 | carnivore | Vari & Hadiaty (2012); A. M. Davis, unpublished data. |
| *Leiopotherapon aheneus* | 0.68 | 67.1 | 149.25 (20) | 1.88 | 145 | 3 | omnivore | Davis *et al*. (2016). |
| *Leiopotherapon macrolepis* | 0.2 | 63.02 | 72.38 (9) | 1.15 | 150 | 1 | omnivore | Le Feuvre et al. (2015), Le Feuvre (2017) |
| *Leiopotherapon plumbeus* | 0.07 | 76.91 | 54.95 (2) | 0.71 | 131 | 2 | carnivore | Davis *et al*. (2016). |
| *Leiopotherapon unicolor* | 0.1 | 139.4 | 127.8 (22) | 0.92 | 330 | 4 | carnivore | Davis *et al*. (2016). |
| *Medialuna californiensis* | 0.963 | 179.5 | 489.4 (1) | 2.73 | 388.32 | 1 | herbivore | Boerger (2011). |
| *Mesopristes argenteus* | 0.04 | 173 | 200 (7) | 1.16 | 300 | 3 | carnivore | Davis *et al*. (2016). |
| *Mesopristes cancellatus* | 0.25 | 115 | 85 (1) | 0.74 | 295 | 2 | omnivore | Davis *et al*. (2016). |
| *Microcanthus strigatus* | 0.55 | 120 | 150 (5) | 1.25 | 161.8 | 3 | omnivore | A. M. Davis, unpublished data. |
| *Neatypus obliquus* | 0.2 | 160 | 226 (2) | 1.41 | 194.16 | 2 | carnivore | A. M. Davis, unpublished data. |
| *Neoscorpis lithophilus* | 0.963 | 145 | 321 (2) | 2.21 | 500 | 2 | herbivore | Joubert & Hanekom (1980). A. M. Davis, unpublished data. |
| *Oplegnathus conwayi* | 0.224 | 252 | 280 (1) | 1.11 | 728.1 | 2 | carnivore | Chater *et al.* (1995). |
| *Oplegnathus fasciatus* | 0.7 | 98 | 135 (1) | 1.38 | 647.2 | 1 | omnivore | A. M. Davis, unpublished data. |
| *Oplegnathus insignis* | 0.001 | 315 | 380 (1) | 1.21 | 533.94 | 1 | carnivore | Medina *et al*. (2004). |
| *Oplegnathus punctatus* | 0.2 | 320 | 400 (1) | 1.25 | 695.74 | 1 | omnivore | A. M. Davis, unpublished data. |
| *Oplegnathus woodwardi* | 0.6 | 310 | 360 (2) | 1.16 | 404.5 | 2 | omnivore | A. M. Davis, unpublished data. |
| *Parascorpis typus* | 0.02 | 200 | 197 (1) | 0.99 | 485.4 | 1 | carnivore | Lechanteur, YARG (1999) |
| *Pelates quadrilineatus* | 0.03 | 128.8 | 121 (8) | 0.94 | 246 | 2 | carnivore | Davis *et al*. (2016). |
| *Pelates sexlineatus* | 0.03 | 118 | 112.9 (16) | 0.96 | 230 | 2 | carnivore | Davis *et al*. (2016). |
| *Pelsartia humeralis* | 0.05 | 153.5 | 141.91 (2) | 0.92 | 312 | 2 | carnivore | Davis *et al*. (2016). |
| *Pingalla gilberti* | 0.84 | 86 | 180 (36) | 2.09 | 105 | 4 | herbivore | Davis *et al*. (2016). |
| *Pingalla lorentzi* | 0.66 | 108.65 | 249.5 (6) | 2.30 | 200 | 3 | omnivore | Davis *et al*. (2016). |
| *Pingalla midgeleyi* | 0.96 | 92.4 | 266.6 (10) | 2.82 | 120 | 4 | herbivore | Davis *et al*. (2016). |
| *Rhyncopelates oxyrhynchus* | 0.001 | 186 | 195 (1) | 1.05 | 305 | 1 | carnivore | Davis *et al*. (2016). |
| *Scorpis aequipinnis* | 0.746 | 248 | 321 (2) | 1.29 | 493.49 | 2 | omnivore | Newman *et al*. (2005). Russell (1983). Shepherd & Edgar (2013). |
| *Scorpis georgiana* | 0.4 | 255 | 388 (3) | 1.52 | 372.14 | 2 | omnivore | A. M. Davis, unpublished data. |
| *Scorpis lineolata* | 0.001 | 295 | 527 (2) | 1.79 | 299.33 | 3 | carnivore | Bulman *et al.* (2000). |
| *Scorpis violacea* | 0.001 | 215 | 330 (3) | 1.53 | 450 | 2 | carnivore | Kingsford and MacDiarmid (1988). A. M. Davis, unpublished data. |
| *Scortum barcoo* | 0.95 | 247 | 1027 (4) | 4.16 | 290 | 2 | herbivore | Davis *et al*. (2016). |
| *Scortum hillii* | 0.98 | 262 | 1365 (8) | 5.21 | 290 | 3 | herbivore | Davis *et al*. (2016). |
| *Scortum neili* | 0.98 | 212 | 858 (1) | 4.05 | 400 | 1 | herbivore | Corbett et al. (2002) |
| *Scortum ogilbyi* | 0.99 | 318.75 | 1650 (30) | 5.18 | 335 | 3 | herbivore | Davis *et al*. (2016). |
| *Scortum parviceps* | 0.92 | 264 | 1342 (36) | 5.08 | 360 | 4 | herbivore | Davis *et al*. (2016). |
| *Syncomistes bonapartensis* | 0.99 | 212.67 | 1093.5 (12) | 5.14 | 280 | 1 | herbivore | Le Feuvre et al. (2015), Le Feuvre (2017) |
| *Syncomistes butleri* | 0.999 | 213.9 | 862.2 (18) | 4.03 | 280 | 3 | herbivore | Davis *et al*. (2016). |
| *Syncomistes kimberleyensis* | 0.99 | 114.3 | 444.3 (5) | 3.89 | 200 | 2 | herbivore | Le Feuvre et al. (2015), Le Feuvre (2017) |
| *Syncomistes rastellus* | 0.95 | 143.78 | 558 (12) | 3.88 | 166 | 2 | herbivore | Davis *et al*. (2016). |
| *Syncomistes versicolor* | 0.97 | 105.0 | 432.0 (4) | 4.11 | 170 | 1 | herbivore | Le Feuvre et al. (2015), Le Feuvre (2017) |
| *Syncomistes wunambul* | 0.99 | 193.1 | 1415.6 (5) | 7.33 | 250 | 1 | herbivore | Le Feuvre et al. (2015), Le Feuvre (2017) |
| *Syncomistes trigonicus* | 0.99 | 102.5 | 500.5 (12) | 4.88 | 120 | 3 | herbivore | Davis *et al*. (2016). |
| *Terapon jarbua* | 0.03 | 152.5 | 143.55 (15) | 0.94 | 295 | 3 | carnivore | Davis *et al*. (2016). |
| *Terapon puta* | 0.05 | 141.72 | 132.13 (5) | 0.93 | 180 | 1 | carnivore | Davis *et al*. (2016). |
| *Terapon theraps* | 0.001 | 148.88 | 144.88 (8) | 0.97 | 300 | 3 | carnivore | Davis *et al*. (2016). |
| *Tilodon sexfasciatum* | 0.39 | 128.8 | 187 (1) | 1.45 | 323.6 | 2 | omnivore | Shepherd & Edgar (2013). |
| *Variichthys lacustris* | 0.54 | 149.73 | 137.72 (11) | 0.92 | 220 | 2 | omnivore | Davis *et al*. (2016). |

**Figure S3** Partial least squares (PLS) regression relationships between diet (proportion of plant material) and intestinal length (a); diet (proportion of plant material) and body shape (raw GPA landmarks) variation (b) and body shape variation (raw GPA landmarks) and intestinal length (c); diet (proportion of plant material) and maximum body size (SL: d); and maximum body size and intestinal length (e); and body centroid size and intestinal length (f). ). Diet and RIL variation color-coded (green = herbivores, red = carnivores and yellow = omnivores) according to broad trophic categories outlined in Table S3.

**Figure S4** Warp grids depicting shape change within each body shape module (head shape: a-b), trunk-abdominal shape (c-d), caudal shape (e-f) that are orthogonal to the axis of integration (PC1 axis extremes for each module). Partial least squares regression relationship between diet (proportion of plant material) and cranial shape module (g). Diet and RIL variation color-coded (green = herbivores, red = carnivores and yellow = omnivores) according to broad trophic categories outlined in Table S3.

**Table S4** Results of Broken-stick criterion test for *geomorph* principal components analysis (*gm.prcomp*) eigenvalues to identify significant body shape PC axes for retention in subsequent analyses (first 10 axes of 30 reported only).

| Principal component (axis) | Eigenvalue | Observed proportion of variance explained | | Broken stick value | Significant |
| --- | --- | --- | --- | --- | --- |
| PC1 | 0.0018 | 0.29 | 0.13 | | TRUE |
| PC2 | 0.0013 | 0.21 | 0.10 | | TRUE |
| PC3 | 0.0009 | 0.15 | 0.08 | | TRUE |
| PC4 | 0.0005 | 0.08 | 0.07 | | TRUE |
| PC5 | 0.0003 | 0.05 | 0.06 | | FALSE |
| PC6 | 0.0002 | 0.04 | 0.06 | | FALSE |
| PC7 | 0.0002 | 0.03 | 0.05 | | FALSE |
| PC8 | 0.0002 | 0.03 | 0.05 | | FALSE |
| PC9 | 0.0001 | 0.02 | 0.04 | | FALSE |
| PC10 | 0.0001 | 0.02 | 0.04 | | FALSE |

**Table S5** Results of model fit testing of and Equal Rates (ER) and All Rates Different (ARD) model of evolution of a binary ‘herbivore’ and ‘non-herbivore’ trait evolution across our 8 tree set (‘fitDiscrete’ function *phytools*). Tip states for species sourced from table S3.

| Model | AICc | (ΔAICc) | Weight |
| --- | --- | --- | --- |
| ER | 82.60 | 0 | 0.54 |
| ARD | 82.93 | 0.33 | 0.46 |

**Table S6** Comparison of PLS integration tests (*phylo.integration* in GEOMORPH) using all 17 Procrustes-aligned species landmarks compared to all 30 body shape axes obtained from a Principal Components Analysis (*gm.prcomp* in GEOMORPH).

| Tree 1 | r-PLS | p-value | Effect Size (Z) |
| --- | --- | --- | --- |
| Body shape PC vs Diet | 0.45 | 0.01 | 2.33 |
| Body shape GPA vs Diet | 0.43 | 0.02 | 2.14 |
| Body shape PC vs Intestine L | 0.46 | 0.00 | 2.53 |
| Body shape GPA vs Intestine L | 0.44 | 0.01 | 2.28 |
| Tree 2 | r-PLS | p-value | Effect Size (Z) |
| Body shape PC vs Diet | 0.44 | 0.01 | 2.19 |
| Body shape GPA vs Diet | 0.43 | 0.02 | 2.16 |
| Body shape PC vs Intestine L | 0.45 | 0.01 | 2.46 |
| Body shape GPA vs Intestine L | 0.44 | 0.01 | 2.27 |
| Tree 3 | r-PLS | p-value | Effect Size (Z) |
| Body shape PC vs Diet | 0.43 | 0.02 | 2.14 |
| Body shape GPA vs Diet | 0.43 | 0.02 | 2.14 |
| Body shape PC vs Intestine L | 0.44 | 0.01 | 2.24 |
| Body shape GPA vs Intestine L | 0.44 | 0.01 | 2.24 |
| Tree 4 | r-PLS | p-value | Effect Size (Z) |
| Body shape PC vs Diet | 0.43 | 0.01 | 2.17 |
| Body shape GPA vs Diet | 0.43 | 0.01 | 2.15 |
| Body shape PC vs Intestine L | 0.45 | 0.01 | 2.41 |
| Body shape GPA vs Intestine L | 0.44 | 0.01 | 2.32 |
| Tree 5 | r-PLS | p-value | Effect Size (Z) |
| Body shape PC vs Diet | 0.41 | 0.02 | 1.97 |
| Body shape GPA vs Diet | 0.43 | 0.01 | 2.19 |
| Body shape PC vs Intestine L | 0.41 | 0.02 | 1.97 |
| Body shape GPA vs Intestine L | 0.44 | 0.01 | 2.28 |
| Tree 6 | r-PLS | p-value | Effect Size (Z) |
| Body shape PC vs Diet | 0.39 | 0.04 | 1.72 |
| Body shape GPA vs Diet | 0.43 | 0.01 | 2.17 |
| Body shape PC vs Intestine L | 0.36 | 0.11 | 1.23 |
| Body shape GPA vs Intestine L | 0.43 | 0.01 | 2.14 |
| Tree 7 | r-PLS | p-value | Effect Size (Z) |
| Body shape PC vs Diet | 0.40 | 0.03 | 1.83 |
| Body shape GPA vs Diet | 0.43 | 0.01 | 2.16 |
| Body shape PC vs Intestine L | 0.36 | 0.10 | 1.32 |
| Body shape GPA vs Intestine L | 0.43 | 0.02 | 2.14 |
| Tree 8 | r-PLS | p-value | Effect Size (Z) |
| Body shape PC vs Diet | 0.38 | 0.05 | 1.64 |
| Body shape GPA vs Diet | 0.43 | 0.01 | 2.21 |
| Body shape PC vs Intestine L | 0.36 | 0.12 | 1.20 |
| Body shape GPA vs Intestine L | 0.43 | 0.01 | 2.18 |

**Table S7** Results of *RRPP* ANOVA tests between body shape (GPA landmarks) and subset of cranial landmarks (#1-4, and 15-17), trunk landmarks (#5, 11-14), and caudal peduncle landmarks (#6-10), respectively (see figure S1) across carnivore, omnivore and herbivore trophic groups (table s3).

| ANOVA | Pairwise test | Distance between group means | UCL -95% confidence limit | Z-score (Standardised Effect size) | p-value |
| --- | --- | --- | --- | --- | --- |
| Overall body shape | carnivore:herbivore | 0.046 | 0.076 | 0.253 | 0.400 |
|  | carnivore:omnivore | 0.025 | 0.062 | -1.164 | 0.876 |
|  | herbivore:omnivore | 0.034 | 0.053 | 0.626 | 0.266 |
| Cranial module | carnivore:herbivore | 0.031 | 0.030 | 1.769 | 0.039 |
|  | carnivore:omnivore | 0.015 | 0.027 | -0.166 | 0.567 |
|  | herbivore:omnivore | 0.022 | 0.022 | 1.674 | 0.051 |
| Trunk module | carnivore:herbivore | 0.031 | 0.033 | 1.388 | 0.084 |
|  | carnivore:omnivore | 0.019 | 0.030 | 0.090 | 0.461 |
|  | herbivore:omnivore | 0.020 | 0.024 | 1.179 | 0.114 |
| Caudal | carnivore:herbivore | 0.014 | 0.021 | 0.415 | 0.341 |
|  | carnivore:omnivore | 0.006 | 0.019 | -1.729 | 0.959 |
|  | herbivore:omnivore | 0.016 | 0.015 | 2.027 | 0.022 |

**Table S8** Results of *r*_PLS_ correlation analysis between morphological PC axes (intestinal length, total body shape, body size (standard length and centroid size) and cranial, trunk and caudal peduncle modules) and diet (logit-transformed proportion of plant material in diet) with statistical significance calculated from 10,000 permutations, conducted on the ML consensus tree. Matrices of subsequent pairwise differences in standardised PLS effects sizes (*z*-Score) for comparisons between each trait or PC axis and associated significance levels of comparisons are also provided. Bold font indicates individual test significance after Benjamini-Hochberg False Discovery Rate (FDR) *p*-value adjustment (Raw *r*_PLS_ values across all 8 trees are available in Tables S9 and S10).

| Test | *r*-PLS | FDR *p*-value | Effect Size (*Z*) | Intestine L vs Diet | Body shape GPA vs Diet | Body shape GPA vs Intestine L | Body size vs Diet | Body size vs Intestine L | Centroid size vs Diet |
| --- | --- | --- | --- | --- | --- | --- | --- | --- | --- |
| Intestine L vs Diet | 0.56 | **0.001** | 5.15 |  |  |  |  |  |  |
| Body shape GPA vs Diet | 0.43 | **0.034** | 2.14 | 1.86 (p=0.17) |  |  |  |  |  |
| Body shape GPA vs Intestine L | 0.44 | **0.028** | 2.28 | 1.76 (p=0.17) | 0.09 (p=0.93) |  |  |  |  |
| Body size vs Diet | 0.02 | 0.898 | 0.23 | **3.61 (p=0.003)** | 1.49 (p=0.23) | 1.59 (p=0.22) |  |  |  |
| Body size vs Intestine L | 0.01 | 0.908 | -0.13 | **3.85 (p=0.002)** | 1.72 (p=0.18) | 1.82 (p=0.17) | 0.25 (p=0.89) |  |  |
| Centroid size vs Diet | 0.05 | 0.800 | 0.46 | **3.53 (p=0.003)** | 1.38 (p=0.25) | 1.48 (p=0.23) | 0.15 (p=0.92) | 0.41 (p=0.88) |  |
| Centroid size vs Intestine L | 0.05 | 0.800 | -0.45 | **4.14 (p<0.001)** | 1.95 (p=0.17) | 2.06 (p=0.17) | 0.48 (p=0.85) | 0.22 (p=0.899) | 0.64 (p=0.74) |
| Test | *r*-PLS | FDR *p*-value | Effect Size (*Z*) | Intestine L vs Diet | Body shape GPA vs Diet | Body shape GPA vs Intestine L | Body size vs Diet | Body size vs Intestine L | Centroid size vs Diet |
| Intestine L vs Diet | 0.56 | **0.001** | 5.15 |  |  |  |  |  |  |
| Head shape vs Diet | 0.50 | **0.001** | 3.48 | 0.31 (p=0.89) |  |  |  |  |  |
| Trunk shape vs Diet | 0.34 | 0.093 | 1.62 | 1.98 (p=0.17) | 1.45 (p=0.23) |  |  |  |  |
| Caudal shape vs Diet | 0.30 | 0.138 | 1.35 | 2.38 (p=0.09) | 1.77 (p=0.17) | 0.27 (p=0.89) |  |  |  |

**Table S9** Raw results (no FDR adjustment) of *r*_PLS_ correlation analysis between morphological PC axes (intestinal length, total body shape, body size (standard length and centroid size) and diet (logit-transformed proportion of plant material in diet) with statistical significance calculated from 10,000 permutations across 8 phylogenetic trees.

|  |  |  |  | Effect sizes for pairwise differences in PLS effect size | | | |  |  |
| --- | --- | --- | --- | --- | --- | --- | --- | --- | --- |
| Tree 1 | *r*-PLS | *p*-value | Effect Size (Z) | Intestine L vs Diet | Body shape GPA vs Diet | Body shape GPA vs Intestine L | Body size vs Diet | Body size vs Intestine L | Centroid size vs Diet |
| Intestine L vs Diet | 0.56 | 0.000 | 5.15 | 0.00 |  |  |  |  |  |
| Body shape GPA vs Diet | 0.43 | 0.015 | 2.14 | 1.86 (P=0.064) | 0.00 |  |  |  |  |
| Body shape GPA vs Intestine L | 0.44 | 0.010 | 2.28 | 1.76 (P=0.078) | 0.09 (P=0.93) | 0.00 |  |  |  |
| Body size vs Diet | 0.02 | 0.816 | 0.23 | 3.61 (P<0.001) | 1.49 (P=0.14) | 1.59 (P=0.11) | 0.00 |  |  |
| Body size vs Intestine L | 0.01 | 0.908 | -0.13 | 3.85 (P<0.001) | 1.72 (P=0.086) | 1.82 (P=0.069) | 0.25 (P=0.80) | 0.00 |  |
| Centroid size vs Diet | 0.05 | 0.644 | 0.46 | 3.53 (P<0.001) | 1.38 (P=0.17) | 1.48 (P=0.14) | 0.15 (P=0.88) | 0.41 (P=0.68) | 0.00 |
| Centroid size vs Intestine L | 0.05 | 0.655 | -0.45 | 4.14 (P<0.001) | 1.95 (P=0.051) | 2.06 (P=0.040) | 0.48 (P=0.63) | 0.22 (P=0.83) | 0.64 (P=0.52) |
| Tree 2 | *r*-PLS | *p*-value | Effect Size (Z) | Intestine L vs Diet | Body shape GPA vs Diet | Body shape GPA vs Intestine L | Body size vs Diet | Body size vs Intestine L | Centroid size vs Diet |
| Intestine L vs Diet | 0.58 | 0.000 | 5.54 | 0.00 |  |  |  |  |  |
| Body shape GPA vs Diet | 0.43 | 0.015 | 2.16 | 2.04 (P=0.042) | 0.00 |  |  |  |  |
| Body shape GPA vs Intestine L | 0.44 | 0.010 | 2.27 | 1.88 (P=0.060) | 0.12 (P=0.90) | 0.00 |  |  |  |
| Body size vs Diet | 0.02 | 0.858 | -0.17 | 4.09 (P<0.001) | 1.75 (P=0.080) | 1.85 (P=0.064) | 0.00 |  |  |
| Body size vs Intestine L | 0.02 | 0.856 | -0.19 | 4.05 (P<0.001) | 1.74 (P=0.081) | 1.85 (P=0.065) | 0.01 (P=0.99) | 0.00 |  |
| Centroid size vs Diet | 0.06 | 0.591 | 0.54 | 3.57 (P<0.001) | 1.28 (P=0.20) | 1.39 (P=0.17) | 0.51 (P=0.61) | 0.51 (P=0.61) | 0.00 |
| Centroid size vs Intestine L | 0.02 | 0.822 | -0.23 | 4.17 (P<0.001) | 1.80 (P=0.071) | 1.91 (P=0.056) | 0.04 (P=0.97) | 0.03 (P=0.98) | 0.55 (P=0.58) |
| Tree 3 | *r*-PLS | *p*-value | Effect Size (Z) | Intestine L vs Diet | Body shape GPA vs Diet | Body shape GPA vs Intestine L | Body size vs Diet | Body size vs Intestine L | Centroid size vs Diet |
| Intestine L vs Diet | 0.54 | 0.000 | 5.19 | 0.00 |  |  |  |  |  |
| Body shape GPA vs Diet | 0.43 | 0.016 | 2.14 | 1.52 (P=0.13) | 0.00 |  |  |  |  |
| Body shape GPA vs Intestine L | 0.44 | 0.012 | 2.24 | 1.48 (P=0.14) | 0.05 (P=0.96) | 0.00 |  |  |  |
| Body size vs Diet | 0.03 | 0.775 | -0.27 | 3.84 (P<0.001) | 1.84 (P=0.065) | 1.92 (P=0.055) | 0.00 |  |  |
| Body size vs Intestine L | 0.05 | 0.611 | -0.50 | 3.98 (P<0.001) | 1.98 (P=0.048) | 2.05 (P=0.040) | 0.17 (P=0.87) | 0.00 |  |
| Centroid size vs Diet | 0.09 | 0.364 | 0.92 | 2.92 (P=0.003) | 1.07 (P=0.28) | 1.14 (P=0.26) | 0.85 (P=0.40) | 1.00 (P=0.32) | 0.00 |
| Centroid size vs Intestine L | 0.06 | 0.571 | -0.58 | 4.09 (P<0.001) | 2.04 (P=0.041) | 2.12 (P=0.034) | 0.21 (P=0.83) | 0.04 (P=0.97) | 1.06 (P=0.29) |
| Tree 4 | *r*-PLS | *p*-value | Effect Size (Z) | Intestine L vs Diet | Body shape GPA vs Diet | Body shape GPA vs Intestine L | Body size vs Diet | Body size vs Intestine L | Centroid size vs Diet |
| Intestine L vs Diet | 0.55 | 0.000 | 5.36 | 0.00 |  |  |  |  |  |
| Body shape GPA vs Diet | 0.43 | 0.014 | 2.15 | 1.54 (P=0.12) | 0.00 |  |  |  |  |
| Body shape GPA vs Intestine L | 0.44 | 0.009 | 2.32 | 1.96 (P=0.051) | 0.19 (P=0.85) | 0.00 |  |  |  |
| Body size vs Diet | 0.02 | 0.819 | -0.23 | 3.95 (P<0.001) | 1.85 (P=0.065) | 1.85 (P=0.064) | 0.00 |  |  |
| Body size vs Intestine L | 0.04 | 0.721 | -0.38 | 4.08 (P<0.001) | 1.94 (P=0.052) | 1.96 (P=0.050) | 0.10 (P=0.92) | 0.00 |  |
| Centroid size vs Diet | 0.05 | 0.624 | 0.48 | 3.33 (P<0.001) | 1.37 (P=0.17) | 1.31 (P=0.19) | 0.51 (P=0.61) | 0.61 (P=0.54) | 0.00 |
| Centroid size vs Intestine L | 0.04 | 0.695 | -0.41 | 4.07 (P<0.001) | 1.95 (P=0.051) | 1.97 (P=0.049) | 0.13 (P=0.90) | 0.03 (P=0.98) | 0.63 (P=0.53) |
| Tree 5 | *r*-PLS | *p*-value | Effect Size (Z) | Intestine L vs Diet | Body shape GPA vs Diet | Body shape GPA vs Intestine L | Body size vs Diet | Body size vs Intestine L | Centroid size vs Diet |
| Intestine L vs Diet | 0.51 | 0.000 | 4.85 | 0.00 |  |  |  |  |  |
| Body shape GPA vs Diet | 0.43 | 0.014 | 2.19 | 1.57 (P=0.12) | 0.00 |  |  |  |  |
| Body shape GPA vs Intestine L | 0.44 | 0.011 | 2.28 | 1.47 (P=0.14) | 0.09 (P=0.93) | 0.00 |  |  |  |
| Body size vs Diet | 0.10 | 0.324 | -0.99 | 4.06 (P<0.001) | 2.28 (P=0.022) | 2.35 (P=0.019) | 0.00 |  |  |
| Body size vs Intestine L | 0.09 | 0.396 | -0.87 | 4.05 (P<0.001) | 2.22 (P=0.027) | 2.29 (P=0.022) | 0.13 (P=0.90) | 0.00 |  |
| Centroid size vs Diet | 0.08 | 0.467 | 0.73 | 2.95 (P=0.003) | 1.17 (P=0.24) | 1.25 (P=0.21) | 1.23 (P=0.22) | 1.13 (P=0.26) | 0.00 |
| Centroid size vs Intestine L | 0.06 | 0.557 | -0.58 | 3.90 (P<0.001) | 2.05 (P=0.040) | 2.12 (P=0.034) | 0.34 (P=0.73) | 0.22 (P=0.83) | 0.93 (P=0.35) |
| Tree 6 | *r*-PLS | *p*-value | Effect Size (Z) | Intestine L vs Diet | Body shape GPA vs Diet | Body shape GPA vs Intestine L | Body size vs Diet | Body size vs Intestine L | Centroid size vs Diet |
| Intestine L vs Diet | 0.53 | 0.000 | 5.08 | 0.00 |  |  |  |  |  |
| Body shape GPA vs Diet | 0.43 | 0.013 | 2.17 | 1.69 (P=0.091) | 0.00 |  |  |  |  |
| Body shape GPA vs Intestine L | 0.43 | 0.015 | 2.14 | 1.62 (P=0.11) | 0.04 (P=0.97) | 0.00 |  |  |  |
| Body size vs Diet | 0.02 | 0.870 | -0.16 | 3.72 (P<0.001) | 1.75 (P=0.080) | 1.75 (P=0.080) | 0.00 |  |  |
| Body size vs Intestine L | 0.04 | 0.710 | -0.40 | 3.91 (P<0.001) | 1.91 (P=0.056) | 1.91 (P=0.056) | 0.17 (P=0.87) | 0.00 |  |
| Centroid size vs Diet | 0.13 | 0.220 | 1.23 | 2.82 (P=0.005) | 0.89 (P=0.38) | 0.90 (P=0.37) | 0.97 (P=0.33) | 1.15 (P=0.25) | 0.00 |
| Centroid size vs Intestine L | 0.05 | 0.626 | -0.50 | 4.09 (P<0.001) | 2.01 (P=0.044) | 2.01 (P=0.045) | 0.22 (P=0.82) | 0.05 (P=0.96) | 1.24 (P=0.22) |
| Tree 7 | *r*-PLS | *p*-value | Effect Size (Z) | Intestine L vs Diet | Body shape GPA vs Diet | Body shape GPA vs Intestine L | Body size vs Diet | Body size vs Intestine L | Centroid size vs Diet |
| Intestine L vs Diet | 0.53 | 0.000 | 5.05 | 0.00 |  |  |  |  |  |
| Body shape GPA vs Diet | 0.43 | 0.014 | 2.16 | 1.54 (P=0.12) | 0.00 |  |  |  |  |
| Body shape GPA vs Intestine L | 0.43 | 0.015 | 2.14 | 1.76 (P=0.078) | 0.14 (P=0.89) | 0.00 |  |  |  |
| Body size vs Diet | 0.01 | 0.885 | -0.13 | 3.69 (P<0.001) | 1.76 (P=0.078) | 1.69 (P=0.090) | 0.00 |  |  |
| Body size vs Intestine L | 0.03 | 0.746 | -0.34 | 3.81 (P<0.001) | 1.88 (P=0.060) | 1.82 (P=0.068) | 0.15 (P=0.88) | 0.00 |  |
| Centroid size vs Diet | 0.14 | 0.179 | 1.34 | 2.62 (P=0.009) | 0.82 (P=0.41) | 0.71 (P=0.48) | 1.05 (P=0.30) | 1.19 (P=0.24) | 0.00 |
| Centroid size vs Intestine L | 0.06 | 0.591 | -0.57 | 4.05 (P<0.001) | 2.05 (P=0.040) | 2.00 (P=0.046) | 0.30 (P=0.77) | 0.14 (P=0.89) | 1.36 (P=0.17) |
| Tree 8 | *r*-PLS | *p*-value | Effect Size (Z) | Intestine L vs Diet | Body shape GPA vs Diet | Body shape GPA vs Intestine L | Body size vs Diet | Body size vs Intestine L | Centroid size vs Diet |
| Intestine L vs Diet | 0.52 | 0.000 | 5.00 | 0.00 |  |  |  |  |  |
| Body shape GPA vs Diet | 0.43 | 0.011 | 2.21 | 1.85 (P=0.065) | 0.00 |  |  |  |  |
| Body shape GPA vs Intestine L | 0.43 | 0.015 | 2.18 | 1.58 (P=0.11) | 0.15 (P=0.88) | 0.00 |  |  |  |
| Body size vs Diet | 0.04 | 0.734 | -0.35 | 3.80 (P<0.001) | 1.85 (P=0.065) | 1.89 (P=0.059) | 0.00 |  |  |
| Body size vs Intestine L | 0.05 | 0.626 | -0.51 | 3.96 (P<0.001) | 1.98 (P=0.048) | 2.01 (P=0.044) | 0.11 (P=0.91) | 0.00 |  |
| Centroid size vs Diet | 0.12 | 0.243 | 1.17 | 2.84 (P=0.005) | 0.87 (P=0.39) | 0.97 (P=0.33) | 1.06 (P=0.29) | 1.18 (P=0.24) | 0.00 |
| Centroid size vs Intestine L | 0.05 | 0.630 | -0.51 | 3.97 (P<0.001) | 1.98 (P=0.048) | 2.01 (P=0.044) | 0.11 (P=0.91) | 0.00 (P=1.00) | 1.19 (P=0.24) |

**Table S10** Raw results (no FDR adjustment) of *r*_PLS_ correlation analysis between morphological PC axes (intestinal length, cranial, trunk and caudal peduncle shape) and diet (logit-transformed proportion of plant material in diet) with statistical significance calculated from 10,000 permutations across 8 phylogenetic trees.

| Tree 1 | *r*_PLS_ | Effect Size (Z) | Intestine L vs Diet | Cranial shape vs Diet | Trunk shape vs Diet | Caudal shape vs Diet |
| --- | --- | --- | --- | --- | --- | --- |
| Intestine L vs diet | 0.56 (p<0.001) | 5.15 | 0 |  |  |  |
| HeadShape vs Diet | 0.50 (p<0.001) | 3.48 | 0.30 (p=0.76) | 0 |  |  |
| TrunkShape vs Diet | 0.34 (p<0.001) | 1.62 | 1.98 (p=0.05) | 1.45 (p=0.15) | 0 |  |
| CaudalShape vs Diet | 0.30 (p=0.08) | 1.35 | 2.38 (p=0.02) | 1.77 (p=0.08) | 0.27 (p=0.78) | 0 |
| Tree 2 | *r*_PLS_ | Effect Size (Z) | Intestine L vs Diet | Cranial shape vs Diet | Trunk shape vs Diet | Caudal shape vs Diet |
| Intestine L vs Diet | 0.58 (p<0.001) | 5.54 | 0 |  |  |  |
| Cranial shape vs Diet | 0.50 (p<0.001) | 3.40 | 0.25 (p=0.80) | 0 |  |  |
| Trunk shape vs Diet | 0.33 (p=0.07) | 1.51 | 2.18 (p=0.03) | 1.57 (p=0.12) | 0 |  |
| Caudal shape vs Diet | 0.30 (p=0.09) | 1.37 | 2.07 (p=0.04) | 1.53 (p=0.13) | 0.04 (p=0.96) | 0 |
| Tree 3 | *r*_PLS_ | Effect Size (Z) | Intestine L vs Diet | Cranial shape vs Diet | Trunk shape vs Diet | Caudal shape vs Diet |
| Intestine L vs Diet | 0.54 (p<0.001) | 4.98 | 0 |  |  |  |
| Cranial shape vs Diet | 0.45 (p=0.001) | 2.98 | 0.49 (p=0.63) | 0 |  |  |
| Trunk shape vs Diet | 0.34 (p=0.05) | 1.73 | 1.95 (p=0.05) | 1.17 (p=0.24) | 0 |  |
| Caudal shape vs Diet | 0.31 (p=0.08) | 1.62 | 1.82 (p=0.07) | 1.11 (p=0.26) | 0.001 (p=1.0) | 0 |
| Tree 4 | *r*_PLS_ | Effect Size (Z) | Intestine L vs Diet | Cranial shape vs Diet | Trunk shape vs Diet | Caudal shape vs Diet |
| Intestine L vs Diet | 0.55 (p<0.001) | 5.09 | 0 |  |  |  |
| Cranial shape vs Diet | 0.49 (p<0.001) | 3.31 | 0.23 (p=0.815) | 0 |  |  |
| Trunk shape vs Diet | 0.33 (p=0.06) | 1.55 | 2.18 (p=0.03) | 1.57 (p=0.11) | 0 |  |
| Caudal shape vs Diet | 0.31 (p=0.08) | 1.38 | 2.06 (p=0.04) | 1.52 (p=0.13) | 0.04 (p=0.97) | 0 |
| Tree 5 | *r*_PLS_ | Effect Size (Z) | Intestine L vs Diet | Cranial shape vs Diet | Trunk shape vs Diet | Caudal shape vs Diet |
| Intestine L vs Diet | 0.51 (p<0.001) | 4.85 | 0 |  |  |  |
| Cranial shape vs Diet | 0.41 (p=0.003) | 2.64 | 0.58 (p=0.56) | 0 |  |  |
| Trunk shape vs Diet | 0.35 (p=0.03) | 1.90 | 1.60 (p=0.11) | 0.80 (p=0.42) | 0 |  |
| Caudal shape vs Diet | 0.33 (p=0.03) | 1.81 | 1.62 (p=0.11) | 0.82 (p=0.41) | 0.03 (p=0.97) | 0 |
| Tree 6 | *r*_PLS_ | Effect Size (Z) | Intestine L vs Diet | Cranial shape vs Diet | Trunk shape vs Diet | Caudal shape vs Diet |
| Intestine L vs Diet | 0.55 (p<0.001) | 5.08 | 0 |  |  |  |
| Cranial shape vs Diet | 0.38 (p=0.01) | 2.33 | 1.33 (p=0.18) | 0 |  |  |
| Trunk shape vs Diet | 0.34 (p=0.04) | 1.81 | 2.03 (p=0.04) | 0.54 (p=0.59) | 0 |  |
| Caudal shape vs Diet | 0.28 (p-0.14) | 1.07 | 2.50 (p=0.01) | 0.99 (p=0.32) | 0.49 (p=0.63) | 0 |
| Tree 7 | *r*_PLS_ | Effect Size (Z) | Intestine L vs Diet | Cranial shape vs Diet | Trunk shape vs Diet | Caudal shape vs Diet |
| Intestine L vs Diet | 0.54 (p<0.001) | 5.05 | 0 |  |  |  |
| Cranial shape vs Diet | 0.39 (p=0.007) | 2.45 | 1.26 (p=0.21) | 0 |  |  |
| Trunk shape vs Diet | 0.35 (p=0.03) | 1.88 | 1.76 (p=0.08) | 0.44 (p=0.66) | 0 |  |
| Caudal shape vs Diet | 0.29 (P=0.12) | 1.11 | 2.3 (p=0.02) | 0.94 (p=0.35) | 0.51 (p=0.61) | 0 |
| Tree 8 | *r*_PLS_ | Effect Size (Z) | Intestine L vs Diet | Cranial shape vs Diet | Trunk shape vs Diet | Caudal shape vs Diet |
| Intestine L vs Diet | 0.53 (p<0.001) | 5.00 | 0 |  |  |  |
| Cranial shape vs Diet | 0.38 (p=0.01) | 2.22 | 1.40 (p=0.16) | 0 |  |  |
| Trunk shape vs Diet | 0.34 (p=0.04) | 1.76 | 1.92 (p=0.06) | 0.42 (p=0.68) | 0 |  |
| Caudal shape vs Diet | 0.28 (p=0.13) | 1.09 | 2.44 (p=0.01) | 0.88 (p=0.38) | 0.48 (p=0.63) | 0 |

**Table S11** AIC weights (AIC_W_) representing the relative ranked likelihood of each of five evolutionary models applied to different elements of Terapontoidei morphology. Lower AIC_C_ scores indicate a better fit, and higher Akaike weights (AIC_W_) indicate better support.

| Variable | Model | AIC | Difference (ΔAICc) | AICw |
| --- | --- | --- | --- | --- |
| Intestinal length | OUDD | -105.59 | 0.00 | 1.00 |
|  | BMDD | -66.33 | 39.27 | 0.00 |
|  | OUDI | -63.39 | 42.21 | 0.00 |
|  | BMDI | -61.85 | 43.74 | 0.00 |
|  | ACDC | -59.72 | 45.88 | 0.00 |
| Body Shape (PC1-4) | Model | AIC | Difference | AICw |
|  | OUDD | -1653.62 | 0.00 | 1.00 |
|  | OUDI | -1625.56 | 28.06 | 0.00 |
|  | BMDD | -1577.37 | 76.26 | 0.00 |
|  | BMDI | -1565.96 | 87.66 | 0.00 |
|  | ACDC | -1557.58 | 96.04 | 0.00 |
| Maximum body size | Model | AIC | Difference | AICw |
|  | OUDI | -54.53 | 0.00 | 0.53 |
|  | OUDD | -54.26 | 0.26 | 0.47 |
|  | BMDI | -39.95 | 14.57 | 0.00 |
|  | BMDD | -38.84 | 15.69 | 0.00 |
|  | ACDC | -37.81 | 16.71 | 0.00 |

**Table S12** AIC weights (AIC_W_) representing the relative ranked likelihood of each of five evolutionary models applied to a Brown Motion (BM) model simulated to PC1-PC4 data range on 100 SIMMAPs simulated on the consensus tree under an Equal Rates (ER) model. Lower AIC_C_ scores indicate a better fit, and higher Akaike weights (AIC_W_) indicate better support.

| Model | AICc | Difference | AICw |
| --- | --- | --- | --- |
| BMDI | -1907.72 | 0.00 | 0.90 |
| ACDC | -1903.40 | 4.32 | 0.10 |
| BMDD | -1889.33 | 18.39 | 0.00 |
| OUDI | -1884.46 | 23.26 | 0.00 |
| OUDD | -1881.74 | 25.98 | 0.00 |

**Table S13** C-t (Time-Slice Convergence Metrics) analyses of multivariate body shape (PC1-PC4) run across eight phylogenetic trees (*calcConvCt* function in *convevol*). Tree 1 is our consensus tree.

| Tree | Metric | Overall Ct | Overall p-value | Weighted overall Ct | Weighted p-value |
| --- | --- | --- | --- | --- | --- |
| Tree 1 | Ct1 | -0.053 | 0.95 | -0.056 | 0.25 |
|  | Ct2 | -0.004 | 1.00 | -0.002 | 0.20 |
|  | Ct3 | -0.019 | 0.94 | -0.015 | 0.14 |
|  | Ct4 | -0.001 | 0.52 | 0.001 | 0.01 |
| Tree 2 | Ct1 | -0.054 | 0.98 | -0.101 | 0.68 |
|  | Ct2 | -0.004 | 1.00 | -0.007 | 0.89 |
|  | Ct3 | -0.021 | 0.98 | -0.035 | 0.51 |
|  | Ct4 | -0.001 | 0.90 | -0.002 | 0.14 |
| Tree 3 | Ct1 | -0.056 | 1.00 | -0.084 | 0.53 |
|  | Ct2 | -0.004 | 1.00 | -0.006 | 0.63 |
|  | Ct3 | -0.021 | 0.99 | -0.029 | 0.37 |
|  | Ct4 | -0.002 | 0.85 | -0.001 | 0.06 |
| Tree 4 | Ct1 | -0.057 | 0.98 | -0.105 | 0.74 |
|  | Ct2 | -0.004 | 0.99 | -0.007 | 0.91 |
|  | Ct3 | -0.021 | 0.99 | -0.034 | 0.57 |
|  | Ct4 | -0.001 | 0.87 | -0.001 | 0.14 |
| Tree 5 | Ct1 | -0.085 | 0.98 | -0.105 | 0.59 |
|  | Ct2 | -0.006 | 1.00 | -0.007 | 0.64 |
|  | Ct3 | -0.028 | 0.97 | -0.033 | 0.36 |
|  | Ct4 | -0.002 | 0.92 | -0.001 | 0.06 |
| Tree 6 | Ct1 | -0.099 | 0.88 | -0.147 | 0.33 |
|  | Ct2 | -0.006 | 0.95 | -0.009 | 0.49 |
|  | Ct3 | -0.034 | 0.87 | -0.047 | 0.29 |
|  | Ct4 | -0.003 | 0.87 | -0.005 | 0.55 |
| Tree 7 | Ct1 | -0.105 | 0.87 | -0.168 | 0.42 |
|  | Ct2 | -0.006 | 0.95 | -0.010 | 0.66 |
|  | Ct3 | -0.036 | 0.88 | -0.053 | 0.35 |
|  | Ct4 | -0.003 | 0.88 | -0.005 | 0.65 |
| Tree 8 | Ct1 | -0.096 | 0.84 | -0.146 | 0.49 |
|  | Ct2 | -0.006 | 0.94 | -0.009 | 0.59 |
|  | Ct3 | -0.032 | 0.77 | -0.048 | 0.38 |
|  | Ct4 | -0.002 | 0.40 | -0.002 | 0.08 |

**Table S14** Results of *RRphlylo* analysis run over collection of eight best trees for convergence in body shape (all PC axes) across specialised herbivores.

| Phylogenetic tree | State assessed | ang.state | ang.state.time | p.ang.state | p.ang.state.time |
| --- | --- | --- | --- | --- | --- |
| Tree 1 | Herbivores | 82.55 | 1.52 | 0.01 | 0.99 |
| Tree 2 | Herbivores | 81.03 | 1.75 | 0.00 | 1.00 |
| Tree 3 | Herbivores | 80.92 | 1.73 | 0.00 | 1.00 |
| Tree 4 | Herbivores | 85.01 | 1.85 | 0.05 | 1.00 |
| Tree 5 | Herbivores | 81.56 | 1.49 | 0.00 | 0.97 |
| Tree 6 | Herbivores | 81.69 | 1.35 | 0.00 | 1.00 |
| Tree 7 | Herbivores | 81.69 | 1.32 | 0.00 | 1.00 |
| Tree 8 | Herbivores | 81.69 | 1.44 | 0.00 | 1.00 |

**Table S15** Results of *ℓ1ou* analysis run over 8 phylogenetic trees for intestinal length residuals.

| Tree | Model | No. of shifts | No. of regimes | Number of clades converging |
| --- | --- | --- | --- | --- |
| 1 | AICc | 14 | 3 | 13 |
|  | pBIC | 5 | 4 | 2 |
| 2 | AICc | 14 | 4 | 14 |
|  | pBIC | 4 | 3 | 2 |
| 3 | AICc | 14 | 5 | 14 |
|  | pBIC | 5 | 4 | 2 |
| 4 | AICc | 14 | 4 | 14 |
|  | pBIC | 4 | 4 | 0 |
| 5 | AICc | 15 | 6 | 14 |
|  | pBIC | 0 | 0 | 0 |
| 6 | AICc | 15 | 6 | 13 |
|  | pBIC | 3 | 3 | 0 |
| 7 | AICc | 15 | 6 | 13 |
|  | pBIC | 2 | 2 | 0 |
| 8 | AICc | 11 | 6 | 8 |
|  | pBIC | 3 | 0 | 0 |

**Figure S5** Convergent shifts in Terapontoidei optimum morphology (body shape PC 1-4) using *ℓ1ou* under a multi-peak Ornstein–Uhlenbeck (OU) process, with regimes selected using AIC_C_ (top panel). Phylogenetic tree, with phenotypic peak shifts from mean trait values indicated by coloured branches, and non-convergent (grey) regimes painted onto the branches (edges of the same colour are inferred to have converged). Bar graphs show the range of the phenotypic values for the trait used in analysis.

**Figure S6** Convergent shifts in Terapontoidei optimum morphology (log10 maximum standard length (mm)) using *ℓ1ou* under a multi-peak Ornstein–Uhlenbeck (OU) process, with regimes selected using AIC_C_. Phylogenetic tree, with phenotypic peak shifts from mean trait values indicated by coloured branches, and non-convergent (grey) regimes painted onto the branches (edges of the same colour are inferred to have converged). Bar graphs show the range of the phenotypic values for the trait used in analysis.

**Supplementary information references.**

Aldana, M., Pulgar, J.J., Ogalde, F. & Ojeda, F.P. (2002). Morphometric and parasitological evidence for ontogenetic and geographical dietary shifts in intertidal fishes. *Bull. Mar. Sci*., 70, 55–74.

Anderson TA. Histological and cytological structure of the gastrointestinal tract of the luderick, *Girella tricuspidata* (pisces, kyphosidae), in relation to diet. J Morphol. 1986 Oct;190(1):109-119. doi: 10.1002/jmor.1051900110. PMID: 29940706.

Barry, J.P. & Ehret, M.J. (1993). Diet, food preference, and algal availability for fishes and crabs on intertidal reef communities in southern California. *Environ. Biol. Fishes*, 37, 75-95.

Behrens MD, Lafferty KD (2012) Geographic Variation in the Diet of Opaleye (*Girella nigricans*) with Respect to Temperature and Habitat. PLoS ONE 7(9): e45901. https://doi.org/10.1371/journal.pone.0045901

Bennett, B.A. & Griffiths, C.L. (1986). Aspects of the biology of galjoen *Coracinus capensis* (Cuvier) off the South-Western Cape, South Africa. *Sth Afr. J. Mar. Sci*., 4, 153-162.

Boerger, C. (2011). *Life History, diet and production of an herbivorous temperate marine fish*, *Medialuna californiensis* (Family: Kyphosidae). Master of Science in Biology. California. California State University Northridge. 33p.

Boyle, K.S. & Horn, M.H. (2006). Comparison of feeding guild structure and ecomorphology of intertidal fish assemblages from central California and central Chile. *Mar. Ecol. Progr. Ser.* 319, 65–84.

Bulman,C., Althaus, F., He, X., Bax N.J. & Williams, A. (2001). Diets and trophic guilds of demersal fishes of the south-eastern Australian shelf. *Mar. Freshwater Res*., 52, 537–48.

Chater, S.A., Ferguson, R. van der Elst, R.P. Govender, A. & Beckley, L.E. (1995). Catch statistics and notes on the biology of two knifejaw species (Teleostei: Oplegnathidae) from Natal, South Africa. *Lammergeyer* 43, 6-14.

Choat, J.H., Clements, K.D. & Robbins, W.D. (2002). The trophic status of herbivorous fishes on coral reefs I. Dietary analysis. *Mar. Biol*., 140, 613–623.

Choat, J.H., Robbins, W.D. & Clements, K.D. The trophic status of herbivorous fishes on coral reefs. Marine Biology 145, 445–454 (2004). https://doi.org/10.1007/s00227-004-1341-7

Clements K. D. & Choat, J.H. (1997). Comparison of herbivory in the closely-related marine fish genera Girella and Kyphosus. *Mar. Biol*., 127, 579-586.

Clements, K.D. & Zemke‐White, W.L. (2008). Diet of subtropical herbivorous fishes in northeastern New Zealand. *New Zealand J. Mar. Freshwater Res*., 42, 47-55.

Corbett, L., Batierham, R., Sewell, S., Welch, M., Richards, G., & Larson, H. K. (2002). The Angalarri grunter, *Scortum neili* Allen, Larson and Midgley (Teleostei: Terapontidae): description of adults and their habitat. The Beagle: Records of the Museums and Art Galleries of the Northern Territory, 18, 57–62. https://search.informit.org/doi/10.3316/

Cruz-Escalona, V.H., Aguila-Ramirez, R.N. & Abilia-Cardenas, L.A. (2009.) Food of *Kyphosus elegans* (Perciformes: Khyposidae) in Loreto, Baja California Sur, Mexico. *GIGIMAR Oceanides*, 24, 55–58.

Davis, A.M., Unmack, P., Vari, R.P. &  Betancur-R, R. (2016). Herbivory promotes dental disparification and macroevolutionary dynamics in grunters (Teleostei: Terapontidae), a freshwater adaptive radiation. *Am. Nat.,* 187, 320–333.

Disalvo, L. H., Randall, John E., and Cea, A. 2007. Stomach contents and feeding observations of some Easter Island fishes. Atoll Research Bulletin, 548 1–22. https://doi.org/10.5479/

Fuentes, H.R. (1982). Feeding habits of *Graus nigra* (Labridae) in coastal waters of Iquique in northern Chile. *Jap. J. Ichthyol*., 29, 95-98.

Fuentes LS, Cancino JM (1990) Cambios morfometricos en el tubo digestivo de juveniles de *Girella laevifrons* (Kyphosidae) en funcion de la dieta y del nivel de replecion. Rev Biolo Mar, Valparaiso 25(2):19–26

Glasby, T.M. & Kingsford, M.J. (1994). *Atypichthys strigatus* (Pisces: Scorpididae): An opportunistic planktivore that responds to benthic disturbances and cleans other fishes. *Aust. J. Ecol*., 19, 385-394.

Hyslop, E. J. (1999). Longitudinal variation in fish species composition in the Angabanga river, Papua New Guinea with observations on the trophic status of certain fish species. *Ecol. Freshw. Fish*, 8, 102. DOI: 10.1111/j.1600-0633.1999.tb00060.x.

Joubert, C.S.W. & Hanekom, P.B. (1980). A study of feeding in some inshore reef fish of the Natal coast, South Africa. *Sth Afr. J. Zool*., 15, 262-274.

Kanda, M. & Yamaoka, K. (1995) Tooth and gut morphology in relation to feeding in three girellid species (Perciformes, Girellidae) from southern Japan. *Neth. J. Zool*., 45, 495–512.

Kiparissis, S., Loukovitis, D. & Batargias, C. (2012). First record of the Bermuda sea chub Kyphosus saltatrix (Pisces: Kyphosidae) in Greek waters. *Mar. Biodiv. Rec*., e11, 1–3. doi: <http://dx.doi.org/>

Kingsford, M. & MacDiarmid, A. (1988). Interrelations between planktivorous reef fish and zooplankton in temperate waters. Marine Ecology-progress Series, 48. 103-117. 10.3354/meps048103.

Knudsen S. (2013). Revision of the fish family Kyphosidae (Teleostei: Perciformes). Zootaxa, 3751:1–101.

Kuiter, R.H. (1993). *Coastal fishes of south-eastern Australia*. University of Hawaii Press. Honolulu, Hawaii. 437 p.

Lechanteur YARG. 1999. The ecology and management of reef fishes in False Bay, southwestern Cape, South Africa. PhD thesis, University of Cape Town, South Africa.

Le Feuvre, M.C., Dempster, T. Shelley, J.D. and Swearer, S.E. (2015). Macroecological relationships reveal conservation hotspots and extinction-prone species in Australia’s freshwater fishes. Global Ecol. Biogeogr. doi: 10.1111/geb.12397

Le Feuvre MC (2017) Triple jeopardy in the tropics: assessing extinction risk in Australia’s freshwater biodiversity hotspot. Diss Univ Melb

Lewis, M.A. (2012). The Life History and Ecology of Bluefish, *Girella cyanea*, at Lord Howe Island. Master of Science Thesis. University of Sydney.

Maciolek, J.A. (1981). Consumer trophic relations in an insular tropical estuary. *Bull. Mar. Sci*., 71, 702-711.

Masuda, H. & Allen, G.R. (1993). Meeresfische der Welt - Groß-Indopazifische Region. Tetra Verlag, Herrenteich, Melle. 528 p.

Medina, M., Araya, M. & Vega, C. (2004). Feeding and trophics relationships of coastal fish off northern Chile. *Invest. Mar., Valparaíso*, 32, 33-47.

Nanake, Y., Suda, Y. & Sano, M. (2011). Food habits of fishes on an exposed sandy beach at Fukiagehama, South-West Kyushu Island, Japan. *Helgol. Mar. Res*. 65, 123–131.

Newman, D.M., Jones, P.L. & Laurenson, L.J. (2005), A preliminary examination of the diet and feeding of sea sweep *Scorpis aequipinnis* (Pisces: Scorpididae) from coastal waters of south-western Victoria, Australia? *Hydrobiologia*, 533, 175–186.

Pusey, B.J., Kennard, M.J. & Arthington, A.H. (2004). Freshwater Fishes of North-eastern Australia. CSIRO Publishing, Melbourne. 645 pp.

Randall, J.E. (1967). Food habits of reef fishes of the West Indies. *Stud. Trop. Oceanogr. Miami*, 5, 665-847.

Resh, V.H., Moser, M. & Poole, M. (1999). Feeding habits of some freshwater fishes in streams of Moorea, French Polynesia. *J. Limnol*., 35, 205–210.

Rimmer, D.W. (1986). Changes in diet and the development of microbial digestion in juvenile buffalo bream, *Kyphosus cornelii*. *Mar. Biol*., 92, 443-448.

Russell, B.C. (1983). The food and feeding habits of rocky reef fish of north‐eastern New Zealand. *New Zealand J. Mar. Freshwater Res*., 17, 121-145.

Shepherd, S. & Edgar, G. (2013). (Editors). Ecology of Australian Temperate Reefs: The Unique South. CSIRO Publishing, 23 Oct 2013.

Saburomaru, T. & Tsukahara, H. (1984). The life history of *Girella punctata* Gray on the northern coast of Fukuoka (Japan). *Sci. Bull. Fat. Agric. Kyushu Univ.* 39, 3548.

Silvano, R.A.M. & Guth, A.Z. (2006). Diet and feeding behavior of *Kyphosus* spp. (Kyphosidae) in a Brazilian subtropical reef. *Brazilian Arch. Biol. Technol*., 49, 623-629.

Sturm, E.A. & Horn, M.H. (1998). Food habits, gut morphology and pH, and assimilation efficiency of the zebraperch *Hermosilla azurea*, an herbivorous kyphosid of temperate marine waters. *Mar. Biol*., 132, 515-522.

Tester, A. & Trefz, S.M. (1954). The Food of the Aholehole, *Kuhlia sandvicensis* (Steindachner), in Hawaiian Waters. *Pacific Sci*. 8, 3-10.

Tester, A. L. & Takata, M. (1953). Contribution to the biology of the aholehole a potential baitfish. Final Report, IRAC grant no. 29. Hawaii Mar. Lab. Contrib. 38: iii + 54.

Vari, P.R.; and Hadiaty, K.R. (2012). The Endemic Sulawesi Fish Genus *Lagusia* (Teleostei: Terapontidae). The Raffles Bulletin of Zoology 60(1): 157–162
